# Textured and Hierarchically Porous Hematite Photoanode for Efficient Hydrogen Production via Photoelectrochemical Hydrazine Oxidation

**DOI:** 10.1007/s40820-025-02045-z

**Published:** 2026-01-08

**Authors:** Runfa Tan, Yoo Jae Jeong, Hyun Soo Han, Samadhan Kapse, Seong Sik Shin, Xiaolin Zheng, In Sun Cho

**Affiliations:** 1 Department of Materials Science & Engineering, Ajou University, Suwon, 16499 Republic of Korea; 2 Department of Energy Systems Research, Ajou University, Suwon, 16499 Republic of Korea; 3 Materials Science and Engineering Program, University of Colorado Boulder, Boulder, CO 80303 USA; 4 SKKU Advanced Institute of Nanotechnology (SAINT) and Department of Nanoengineering, Sungkyunkwan University, Suwon, 16419 Republic of Korea; 5 Department of Energy Science, Sungkyunkwan University, Suwon, 16419 Republic of Korea; 6 Department of Mechanical Engineering, Stanford University, Stanford, CA 94305 USA

**Keywords:** Hematite, Hierarchically porous, Texture, Hydrazine oxidation reaction, Solar-to-hydrogen

## Abstract

The performance of hematite (α-Fe_2_O_3_) photoanodes for photoelectrochemical (PEC) water splitting has been limited to around 2–5 mA cm^−2^ under standard conditions due to their short hole diffusion length and sluggish oxygen evolution reaction kinetics. This work overcomes those challenges through a synergistic strategy that co-designs the hematite architecture and the surface reaction pathway. We introduce a textured and hierarchically porous Ti-doped Fe_2_O_3_ (tp-Fe_2_O_3_) photoanode, synthesized via multi-cycle growth and flame annealing method. This unique architecture features a high texture (110), enlarged surface area, and hierarchically porous structure, which enable significantly enhanced bulk charge transport and interfacial charge transfer compared to typical nanorod Ti-doped Fe_2_O_3_ (nr-Fe_2_O_3_). As a result, the tp-Fe_2_O_3_ photoanode achieves a photocurrent density of 3.1 mA cm^−2^ at 1.23 V vs. RHE with exceptional stability over 105 h, notably without any co-catalyst. By replacing the OER with the hydrazine oxidation reaction, the photocurrent further reaches a record-high level of 7.1 mA cm^−2^ at 1.23 V_RHE_. Finally, when we integrate the tp-Fe_2_O_3_ with a commercial Si solar cell, it achieves a solar-to-hydrogen efficiency of 8.7%—the highest reported value for any Fe_2_O_3_-based PV-tandem system. This work provides critical insights into rational Fe_2_O_3_ photoanode design and highlights the potential of hydrazine as an efficient alternative anodic reaction, enabling waste valorization.

**Supplementary Information:**

The online version contains supplementary material available at 10.1007/s40820-025-02045-z.

## Article Highights

- A multi-cycle growth and flame annealing strategy was developed to construct textured and hierarchically porous Ti-doped hematite (tp-Fe_2_O_3_) photoanodes with enhanced charge transport and surface kinetics.
- The hydrazine oxidation reaction was introduced as a fast and thermodynamically favorable alternative to the oxygen evolution reaction, enabling the simultaneous production of hydrogen and the remediation of toxic hydrazine.
- The tp-Fe_2_O_3_-based bias-free photovoltaic-photoelectrochemical tandem device achieved a record solar-to-hydrogen efficiency of 8.7%, demonstrating excellent stability and scalability for sustainable solar fuel generation.

**Supplementary Information:**

The online version contains supplementary material available at 10.1007/s40820-025-02045-z.

## Graphical abstract

**Figure:**
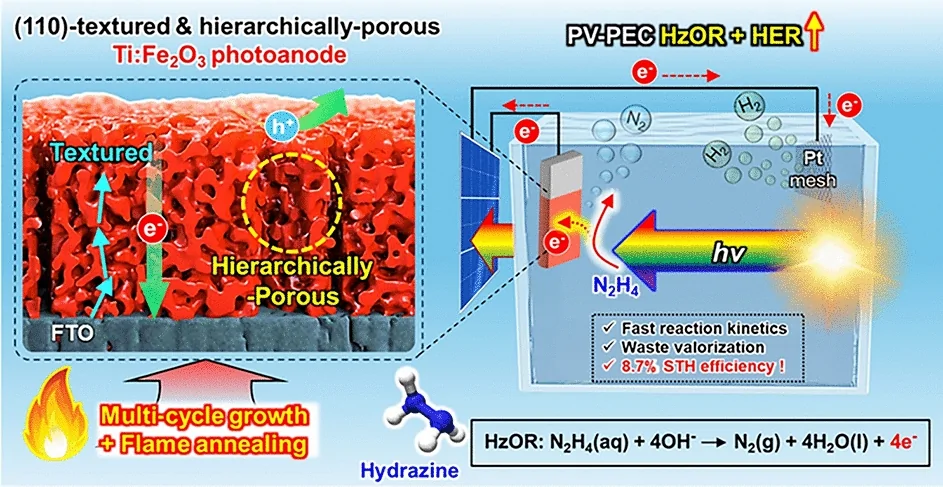

## Introduction

Photoelectrochemical (PEC) water splitting, *i.e.*, direct conversion of sunlight into hydrogen [1–3], faces two major challenges: a lack of suitable photoanode materials and the sluggish kinetics of the oxygen evolution reaction (OER). These limitations result in low photocurrent densities and low solar-to-hydrogen (STH) conversion efficiency of PEC systems [4]. To overcome these challenges, prior research has focused on two complementary strategies: (i) rational design and optimization of photoanode materials for better charge separation and transport [5–7], and (ii) utilizing alternative oxidation reactions (AORs) that have faster kinetics and lower overpotentials than OER [8]. Using AORs not only enhances hydrogen production but also enables the generation of valuable chemicals or the degradation of hazardous pollutants, thereby contributing to environmental remediation and the circular utilization of resources [9–19].

In terms of potential photoanode candidates for PEC systems, hematite (α-Fe_2_O_3_) remains a stronger contender due to its abundance in the Earth’s crust, low cost, excellent chemical stability, and a suitable bandgap (~ 2.1 eV) for efficient visible light absorption [20]. However, Fe_2_O_3_ possesses intrinsic drawbacks, including an extremely short hole diffusion length (~ 2–4 nm), low electrical conductivity, and significant charge recombination in both the bulk and at the surface [21], so the photocurrent densities of bare Fe_2_O_3_ photoanodes typically are below a few mA cm^−2^ at 1.23 V vs. the reversible hydrogen electrode (RHE, V_RHE_) under OER conditions. Although numerous strategies, such as elemental doping, co-catalyst loading, junction formation, and morphological engineering [22–26], have been explored to overcome these limitations, the photocurrent densities achieved by a single hematite photoanode still generally limited to 2–5 mA cm^−2^ at 1.23 V_RHE_, which is still far below the theoretical maximum of 12.6 mA cm^−2^ evaluated based on light absorption. Device-level approaches, such as parallel stacking of multiple transparent hematite photoanodes, have achieved photocurrents approaching ~ 10 mA cm^−2^ under standard conditions [27]; however, these configurations do not represent the intrinsic performance of a single hematite photoanode.

In the context of AORs, only a limited number of studies have explored their application on Fe_2_O_3_ photoanodes, including the oxidation of ethylene glycol, glucose, methanol, and ethanol to replace the sluggish OER [28–31]. Among these, L-cysteine oxidation has shown the highest photocurrent density, reaching up to 4.6 mA cm^−2^ at 1.23 V_RHE_ using a Ti/Si dual-doped porous nanorod Fe_2_O_3_ [32]. However, this value remains significantly below the theoretical maximum value. Moreover, most organic AORs involve complex multi-electron pathways that generate various byproducts, often causing catalyst deactivation, surface fouling, and poor long-term stability. Hydrazine (N_2_H_4_), widely used as a high-energy propellant in aerospace and as a reducing agent in pharmaceutical synthesis, is also highly toxic and carcinogenic; its uncontrolled release into industrial wastewater poses serious environmental risks [33]. Conventional removal of hydrazine waste typically relies on chemical oxidation, which consumes additional reagents and may pose safety concerns. In contrast, hydrazine undergoes a clean and rapid oxidation reaction with minimal formation of byproducts. Therefore, integrating the hydrazine oxidation reaction (HzOR) into PEC systems for hydrogen production offers a dual benefit: (i) providing a thermodynamically and kinetically favorable alternative to the sluggish OER, and (ii) enabling simultaneous H_2_ generation and environmental remediation. These attributes make HzOR an attractive strategy for enhancing the PEC efficiency of Fe_2_O_3_ photoanodes, especially when combined with structural and electronic optimizations.

In this study, we demonstrate a dual strategy to enhance the PEC performance of Fe_2_O_3_ photoanode: (i) structural engineering to improve charge dynamics, and (ii) employing hydrazine oxidation reaction (HzOR) as a kinetically favorable alternative to the sluggish OER. For structural engineering, we developed a novel multi-cycle growth and flame annealing (MGFA) method to fabricate textured, hierarchically porous hematite (tp-Fe_2_O_3_) photoanodes, overcoming the limitations of typical nanorod-structured Fe_2_O_3_ (nr-Fe_2_O_3_). The tp-Fe_2_O_3_ exhibits several key features, including an enhanced (110) crystallographic texture, a uniform depth profile of Ti dopants, and a hierarchically porous architecture with an increased surface area. These structural and compositional modifications significantly enhance charge carrier dynamics, leading to efficient bulk charge transport and interfacial charge transfer. As a result, the tp-Fe_2_O_3_ photoanode exhibits excellent PEC performance, achieving a stable photocurrent density of 3.1 mA cm^−2^ at 1.23 V_RHE_ for OER without any co-catalysts. Notably, by replacing OER with HzOR, a record-high photocurrent density of 7.1 mA cm^−2^ is achieved at the same potential. Furthermore, integration of the tp-Fe_2_O_3_ into a bias-free photovoltaic-photoelectrochemical (PV-PEC) tandem system coupling HzOR with hydrogen evolution reaction (HER) results in a solar-to-hydrogen (STH) efficiency of 8.7%—the highest reported for Fe_2_O_3_-based tandem devices. This work not only provides critical insights into the rational design of high-performance Fe_2_O_3_ photoanodes but also highlights the significant potential of hydrazine oxidation as a sustainable, efficient alternative pathway for solar-driven H_2_ production.

## Experimental Section

### Synthesis of Textured, Hierarchically Porous Hematite (tp-Fe_2_O_3_)

We synthesized tp-Fe_2_O_3_ photoanodes using a MGFA method. Initially, a precursor solution containing 10 mmol of FeCl_3_·6H_2_O, 15 mmol of urea, 1 mmol of TiCl_3_, and 100 mL of deionized water was prepared in a Pyrex glass bottle. Cleaned FTO substrates (1.5 cm × 2.5 cm) were vertically suspended in this solution using Teflon threads and heated at 100 °C for 6 h in a sealed glass bottle to form a Ti-doped FeOOH. Subsequently, multi-cycle growth was performed under the same chemical conditions, but with a shortened reaction time of 2 h per cycle. The growth cycle was repeated six times to optimize the film thickness and hierarchical morphology. The resulting Ti-doped FeOOH films with multi-branched structure were then subjected to flame annealing using a premixed CH_4_/air flame (equivalence ratio Φ = 0.7) for 5 min. The flame annealing process, characterized by an ultrafast heating rate (> 100 °C s^−1^), enables rapid phase transition and crystallization of the Ti-doped FeOOH multi-branched nanorods into Ti-doped Fe_2_O_3_ and dopant activation without severe damage to underlying FTO substrates [34, 35]. The ultrafast heating rate and short duration suppress grain growth. Notably, the flame annealing preserved the multi-branched morphology and induced oriented crystallization of the branches along the core nanorods, promoting a hierarchically porous and (110)-textured structure via epitaxial crystallization from the underlying 1D framework.

### Synthesis of Typical Nanorod-Structured Hematite (nr-Fe_2_O_3_)

We prepared the control nr-Fe_2_O_3_ photoanodes by a single-cycle growth followed by ex situ Ti doping [36]. A precursor solution identical to that used for tp-Fe_2_O_3_, except without Ti addition, was prepared, and the FTO substrates were immersed vertically and heated at 100 °C for 6 h to grow vertically aligned FeOOH nanorods. After drying, Ti doping was performed by dip-coating the FeOOH nanorod film with a 0.02 M Ti(OBu)_4_ solution in 2-methoxyethanol. The films were dried at 80 °C in air and then annealed using the same flame treatment protocol as above to obtain a Ti-doped Fe_2_O_3_ nanorod structure.

### Material Characterization

The crystal structures of the samples were characterized by X-ray diffraction (XRD, Rigaku D/MAX-Ultima III) using Cu Kα radiation (λ = 1.5406 Å). Raman spectra were collected using a confocal Raman microscope (WITec alpha300 R) with a 532 nm laser. Surface morphology and film thickness were examined using field-emission scanning electron microscopy (FE-SEM, JEOL JSM-IT500HR). The detailed internal microstructure and porosity were analyzed using transmission electron microscopy (TEM, FEI Titan 80–300 kV) and energy-dispersive X-ray spectroscopy (EDS). The specific surface area and Barrett–Joyner–Halenda (BJH) pore size distribution were evaluated using Brunauer–Emmett–Teller (BET) analysis using N_2_ adsorption–desorption isotherms (Micromeritics ASAP 2020 Plus). UV–Vis absorption spectra were obtained with a Shimadzu UV-2600i spectrophotometer. Wettability was assessed using a contact angle goniometer (Theta Lite TL101). Surface potential distributions under dark and illuminated conditions were examined via Kelvin probe force microscopy (KPFM, Asylum MFP-30). Chemical states and dopant profiles were analyzed using X-ray photoelectron spectroscopy (XPS, Thermo ESCALAB 250Xi), with Ar^+^ ion etching employed for depth profiling.

### Photoelectrochemical and Electrochemical Measurements

PEC measurements were conducted in a three-electrode configuration under AM 1.5G illumination (1 sun, 100 mW cm^−2^). A 1 M NaOH (pH 13.6) solution was used as the electrolyte for water oxidation. For the HzOR, 0.1 M N_2_H_4_ was added to the 1 M NaOH electrolyte. J–V curves were obtained under dark and 1 sun illumination by linear sweep voltammetry (LSV) at a scan rate of 50 mV s^−1^. Temperature-dependent J–V measurements were performed to derive activation energies based on Arrhenius plots of ln(J) vs. 1/T. Mott–Schottky plots were measured at 1 kHz in the dark to determine the flat-band potential and donor density. Electrochemical impedance spectroscopy (EIS) was performed under potentiostatic conditions over a frequency range of 0.1 Hz to 1 MHz. An equivalent circuit model was employed to extract parameters, including R_bulk_, C_bulk_, R_ct_, and C_trap_. The electrochemically active surface area (ECSA) was estimated from the linear relationship between the capacitive current and the scan rate under dark conditions. Charge transport and transfer efficiencies of the Fe_2_O_3_ photoanode were evaluated as a function of applied potential using H_2_O_2_ as a sacrificial agent. For tandem PV-PEC measurements, a commercial crystalline Si solar cell (Ningbo Aike Electronics Technology Co., Ltd.) was coupled to the PEC cell in a two-electrode configuration. Both devices were simultaneously illuminated to evaluate the feasibility of bias-free H_2_ production via hydrazine oxidation.

## Results and Discussion

### Synthesis and Structural Characterization of tp-Fe_2_O_3_

**Fig. 1:**
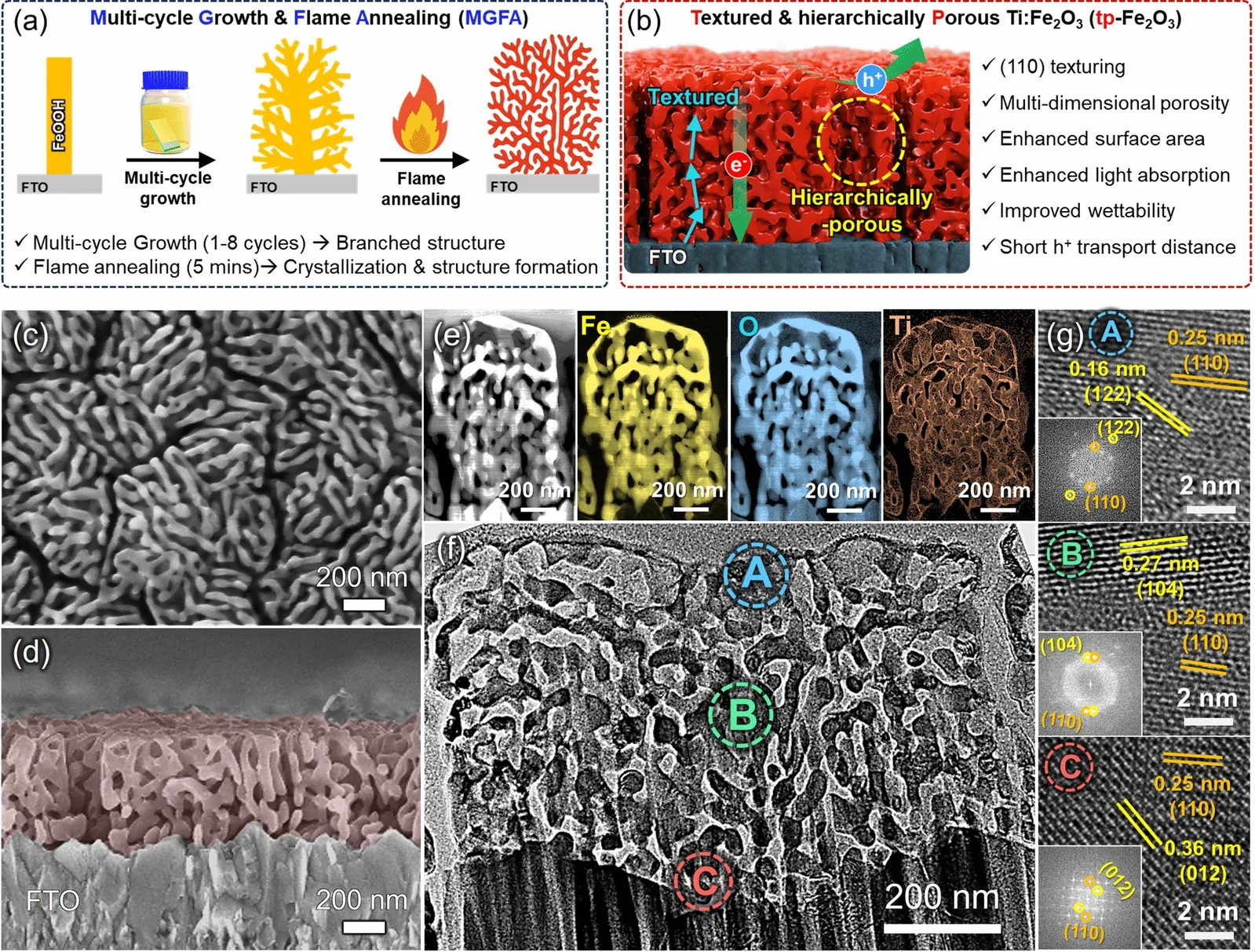
Synthesis and structural features of tp-Fe_2_O_3_ via a MGFA method.** a** Schematic of the MGFA process. **b** Conceptual illustration of tp-Fe_2_O_3_ photoanode and its unique properties. **c, d** Top and cross-sectional SEM images of the synthesized tp-Fe_2_O_3_ photoanode. **e** TEM elemental mapping images via energy-dispersive spectroscopy (EDS) analysis. **f** Cross-sectional and **g** high-resolution TEM images with corresponding  Fast Fourier Transform (FFT) patterns

Figure 1a, b provides a schematic representation of the synthetic process and structural features of textured and hierarchically porous Ti-doped Fe_2_O_3_ (tp-Fe_2_O_3_) obtained via a multi-cycle growth and flame annealing (MGFA) method. Detailed flame setup and method are shown in Figs. S1 and S2. For comparison, the typical nanorod-structured Ti-doped Fe_2_O_3_ (nr-Fe_2_O_3_) was also synthesized (Figs. S3–S5).

Notably, this multi-cycle growth (1–8 cycles, Fig. S6) ensures adequate film thickness and promotes the formation of a cauliflower-like, branched FeOOH morphology with high structural complexity (Fig. S7). The final step involves high-temperature flame annealing under fuel-lean conditions (oxidizing atmosphere) for 5 min, which induces a phase transition and fusion of the branched nanorods into a unique, textured, and hierarchically porous Fe_2_O_3_ (Figs. S8 and S9). A 5 min oxidation flame annealing was identified as optimal, providing the highest porosity and smallest lateral grain size while minimizing FTO substrate damage and achieving the highest photocurrent density for water oxidation (Figs. S10 and S11). To identify the impact of flame annealing, the multi-cycle-grown FeOOH was subjected to conventional furnace annealing for the same duration (5 min). Unlike the flame-treated sample, the conventional furnace-annealed Fe_2_O_3_ exhibited significant aggregation, enlarged grain size, and diminished porosity, resulting in inferior PEC performance (Fig. S12). Therefore, flame annealing is advantageous for constructing a hierarchically porous and network structure of Fe_2_O_3_, which is attributed to its distinct heat transfer mechanisms (directional heat transfer, high heat flux, high temperature, and rapid ramping rate) [36–38].

Figure 1c, d presents top-view and cross-sectional SEM images of the synthesized tp-Fe_2_O_3_ photoanode, respectively. The top-view image (Fig. 1c) reveals a porous and interconnected three-dimensional (3D) network morphology that has a large porosity and surface area. The cross-sectional SEM image (Fig. 1d) displays a well-defined 3D morphology and intimate, uniform deposition on the FTO substrate. Notably, the intimate contact between the tp-Fe_2_O_3_ photoanode and the FTO substrate ensures strong adhesion and good electrical connectivity, which is essential for efficient and stable PEC performance [39, 40]. As shown in Fig. 1e, all three elements (Fe, Ti, and O) are uniformly distributed throughout the nanostructure, indicating homogeneous doping and consistent growth across the film.

Figure 1f shows a cross-sectional view of a TEM image of tp-Fe_2_O_3_, revealing the presence of abundant pores throughout the structure. A higher magnification view further confirms the existence of both nano- and mesopores, highlighting the multi-scale porous architecture (Fig. S13). The hierarchically porous network structure indicates a high degree of structural complexity, which facilitates electrolyte penetration and increases the active surface area, beneficial for the PEC performance [41–43].

Figure 1g presents high-resolution TEM (HR-TEM) images of three selected regions—top surface (A), middle bulk (B), and bottom interface (C)—of the tp-Fe_2_O_3_ photoanode, as marked in Fig. 1f. The corresponding fast Fourier transform (FFT) patterns confirm the high crystallinity and phase purity of Fe_2_O_3_ throughout the film. The observed lattice spacings of 0.16, 0.27, and 0.36 nm correspond to the (122), (104), and (012) planes of α-Fe_2_O_3_, respectively, further validating the well-crystallized structure. Notably, lattice fringes with a spacing of 0.25 nm, corresponding to the (110) planes, are consistently observed in vertical orientation in all three regions, indicating a preferential (110) orientation (i.e., texture) across different regions of the tp-Fe_2_O_3_ film [44, 45].

### Crystal Structure, Composition, Optical, and Wettability Analysis

**Fig. 2:**
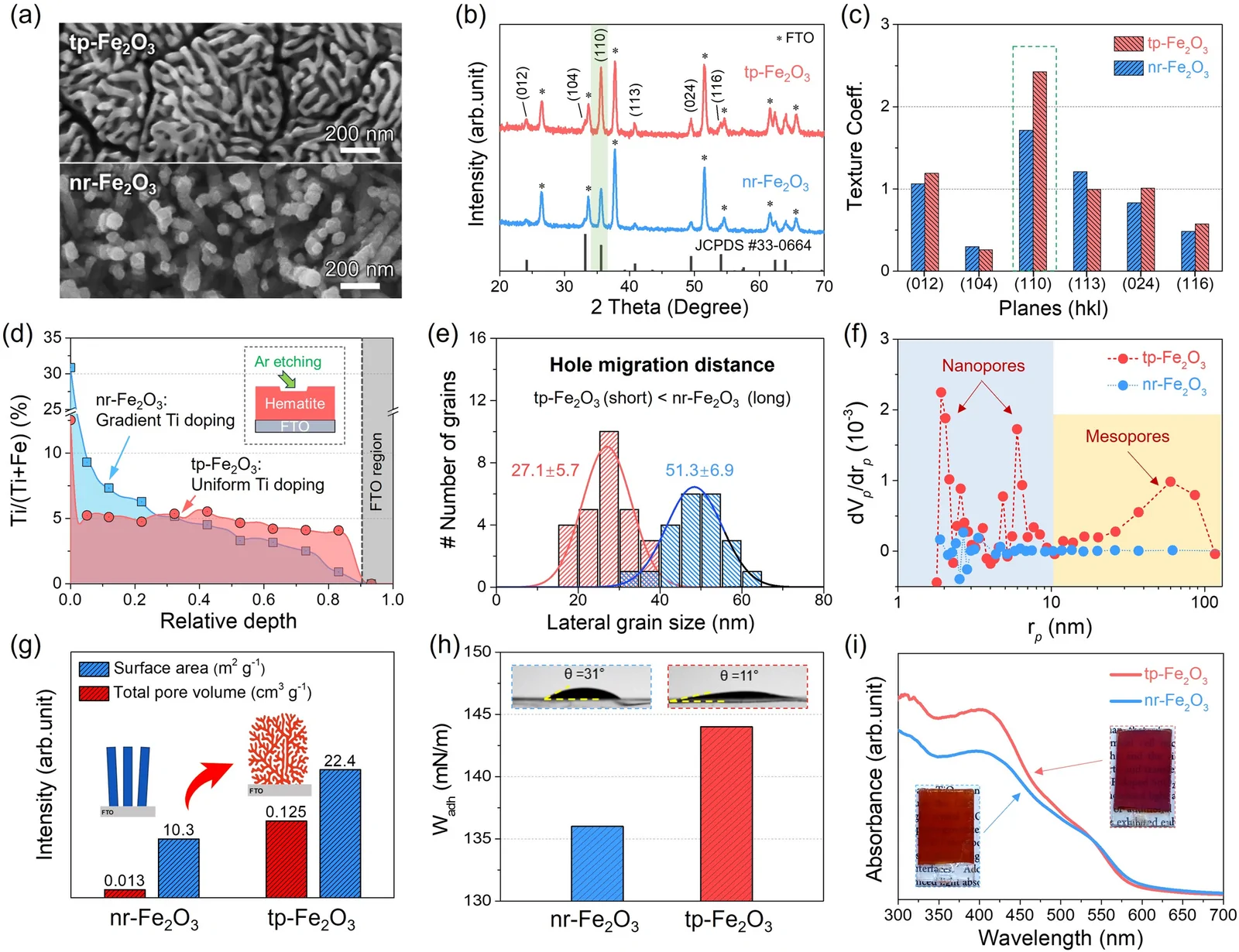
Comparative physicochemical properties of tp-Fe_2_O_3_ and nr-Fe_2_O_3_ photoanodes.** a** Top-view SEM images. **b** XRD pattern and **c** TC analysis. **d** Ti dopant depth profile. **e** Lateral grain size distribution. **f** BJH pore size distribution. **g** Comparison of BET surface area and total pore volume. **h** Contact angle and work of adhesion analyses. **i** UV–Vis absorbance spectra. The inset shows photographs of tp-Fe_2_O_3_ and nr-Fe_2_O_3_ photoanodes

tp-Fe_2_O_3_ exhibits a distinct morphology from the typical nr-Fe_2_O_3_ (Fig. 2a), and we systematically compared their physicochemical properties. First, XRD analysis revealed that both tp-Fe_2_O_3_ and nr-Fe_2_O_3_ are consistent with the standard α-Fe_2_O_3_ phase (JCPDS #33–0664) and both exhibit a stronger (110) diffraction peak, indicating their preferential (110) orientation (Fig. 2b). The texture coefficient (TC) analysis (Fig. 2c) shows that both Fe_2_O_3_ display elevated TC values for the (110) plane compared to other planes. This is attributed to lattice matching between FeOOH and the underlying FTO substrate [46–49], as the substrate directs the crystallographic alignment during the growth. This substrate effect is further supported by a control sample deposited on quartz (Fig. S14). Although a comparable porous structure was formed under the same flame annealing conditions, no (110) texture enhancement was observed. Furthermore, the TC value for tp-Fe_2_O_3_ is much higher than that of nr-Fe_2_O_3_, indicating that the multi-cycle growth process further promotes (110) texturing. This is evidenced by the enhanced (110) diffraction peak intensity and the progressive increase in TC with the increasing growth cycles in tp-Fe_2_O_3_ (Fig. S15). The progressive enhancement of (110) texturing with successive growth cycles is likely driven by the inherently lower surface energy of the (110) plane and its superior lattice compatibility with the FTO substrate, both of which render this orientation thermodynamically favorable and increasingly reinforced during repeated deposition [50, 51]. Based on previous literature, the (110) oriented facet in Fe_2_O_3_ is beneficial for the electron transport, facilitating charge separation and surface redox reactions [44].

To gain compositional insights across the depth of the Fe_2_O_3_ photoanodes, XPS depth profile analysis was performed on both tp-Fe_2_O_3_ and nr-Fe_2_O_3_ with a comparable film thickness of 550 nm (Figs. 2d, S16 and S17). For the nr-Fe_2_O_3_, the Ti dopant concentration gradually decreased from the surface to the bottom (gradient Ti doping). Conversely, the Sn content progressively increased toward the bottom, which was attributed to unintentional Sn diffusion from the underlying FTO substrate. Although Sn diffusion from the FTO substrate can increase carrier density, excessive incorporation may introduce trap sites, leading to recombination and impaired charge transport. In contrast, the tp-Fe_2_O_3_ displayed a uniform Ti doping profile throughout the film and a significantly lower Sn concentration at the bottom. Uniform Ti distribution in the Fe_2_O_3_ ensures consistent bulk conductivity and a stable internal electric field, promoting charge separation and reducing recombination. Consequently, charge transport to the photoanode surface is enhanced.

Figure 2e illustrates the lateral grain size distribution of tp-Fe_2_O_3_ and nr-Fe_2_O_3_ photoanodes (obtained from SEM and TEM images), providing insight into the microstructural characteristics of the synthesized Fe_2_O_3_. The average lateral grain size of tp-Fe_2_O_3_ is determined to be approximately 27.1 ± 5.7 nm, while the average size for nr-Fe_2_O_3_ is two times larger, around 51.3 ± 6.9 nm. The lateral grain size distribution affects the electronic properties of the photoanodes; The reduced grain size in tp-Fe_2_O_3_ leads to a higher density of grain boundaries and enlarged surface area, which facilitates the formation of multiple surface-confined electric fields that enhance charge separation and suppress recombination [43]. Moreover, the reduced lateral grain size minimizes the carrier transport distance to the surface, which is particularly beneficial for Fe_2_O_3_ due to its intrinsically short hole diffusion length (2–4 nm) [20, 25, 43]. In addition, Raman analysis (Fig. S18) reveals that tp-Fe_2_O_3_ has a smaller normalized E_u_ peak intensity (*I*_Eu_/*I*_A1g_), indicating its enhanced structural order, better-preserved lattice symmetry, and lower strain [52–54]. Overall, the structure of tp-Fe_2_O_3_ ensures that photogenerated carriers are efficiently transported to the reactive surface, improving the overall PEC performance.

We analyzed the pore size distribution and specific surface area of tp-Fe_2_O_3_ and nr-Fe_2_O_3_ by the BET method (Fig. S19). As shown in Fig. 2f, tp-Fe_2_O_3_ exhibits a hierarchically porous structure with mesopores and nanopores (< 10 nm), whereas nr-Fe_2_O_3_ contains few nanopores and negligible mesoporosity. Correspondingly, the tp-Fe_2_O_3_ possesses nearly twice the specific surface area and over ten times the total pore volume relative to nr-Fe_2_O_3_ (Fig. 2g). On the other hand, as shown in Fig. 2h, tp-Fe_2_O_3_ exhibits a significantly lower contact angle (11°) than nr-Fe_2_O_3_ (31°), indicating high surface wettability. This improved wettability is attributed to the hierarchically porous structure, which facilitates capillary-driven electrolyte infiltration and increases the effective contact area [55, 56]. The higher work of adhesion observed for tp-Fe_2_O_3_ further confirms its enhanced surface affinity with the electrolyte, thereby promoting more efficient interfacial charge transfer during PEC reactions. Finally, the optical properties of tp-Fe_2_O_3_ and nr-Fe_2_O_3_ photoanodes with similar film thickness (~ 550 nm) were analyzed (Fig. 2i). UV–Vis absorbance measurements reveal that tp-Fe_2_O_3_ exhibits noticeably higher absorbance across the 300–600 nm range compared to nr-Fe_2_O_3_. This enhancement is attributed to its hierarchically porous morphology, which promotes light scattering and internal reflection, thereby extending the optical path length and improving photon capture efficiency [57, 58].

In summary, the tp-Fe_2_O_3_ photoanode prepared by the MGFA method, compared to typical nr-Fe_2_O_3_, has a hierarchically porous architecture, a higher degree of (110) texture, homogeneous dopant distribution, and an enlarged surface area, as well as superior wettability (Fig. S20). These integrated properties are expected to enhance light absorption, facilitate reactant diffusion, and provide abundant active sites, which are highly beneficial for PEC reactions.

### Bulk and Interfacial Charge Carrier Dynamics Analysis

**Fig. 3:**
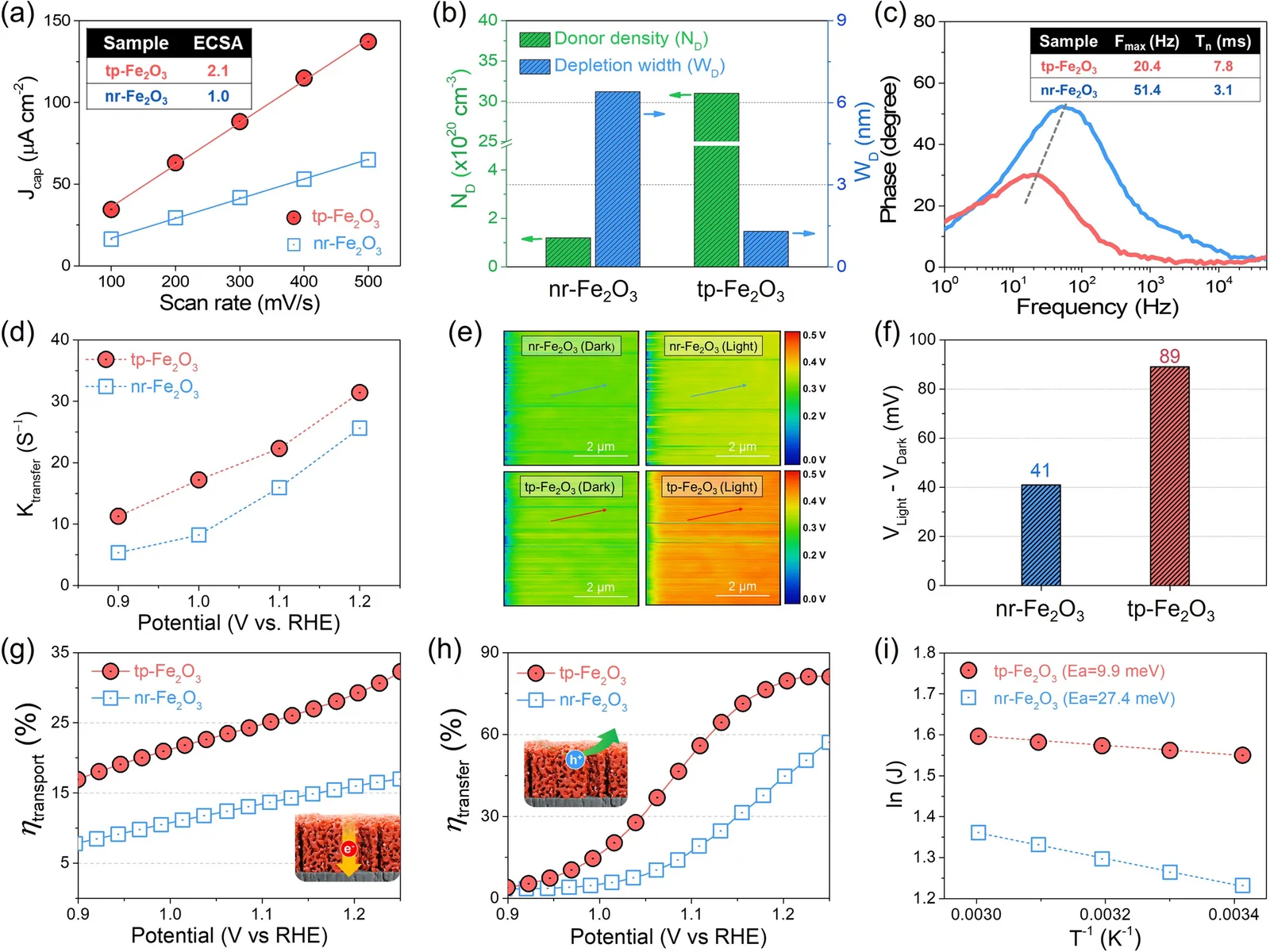
Bulk and interfacial charge carrier dynamics of tp-Fe_2_O_3_ and nr-Fe_2_O_3_ photoanodes. **a** Electrochemical active surface area (ECSA) comparison. **b** Donor density (N_D_) and depletion layer width (W_D_) derived from the Mott–Schottky measurement. **c** Bode phase plot. **d** Surface charge transfer rate constant (K_transfer_ = 1/(R_ct_ × C_trap_), R_ct_ represents the resistance between the photoanode and the electrolyte, and C_trap_ is the capacitance associated with charge accumulation on the surface states. **e** Surface potential distribution and **f** illumination-induced shift (ΔV) from Kelvin probe force microscopy (KPFM). **g** Charge transport and **h** transfer efficiencies. **i** Arrhenius plots of ln(J) vs. 1/T at 1.6 V_RHE_ and corresponding activation energies

We further investigated the impact of structural and compositional differences between tp-Fe_2_O_3_ and nr-Fe_2_O_3_ photoanodes on both bulk and interfacial properties through a series of charge dynamics measurements. First, the electrochemically active surface area (ECSA), which reflects the number of accessible surface sites for interfacial charge transfer (Fig. 3a) [59, 60], was assessed by recording the capacitive current density at various scan rates (Fig. S21). The slope of the tp-Fe_2_O_3_ curve is 2.1 times that of the nr-Fe_2_O_3_; accordingly, its ECSA value is 2.1 times higher, caused by its hierarchically porous structure. Second, the donor density (N_D_) and depletion layer width (W_D_) for tp-Fe_2_O_3_ and nr-Fe_2_O_3_ were determined from the Mott–Schottky plots (Fig. S22). tp-Fe_2_O_3_ exhibits about twenty-four times higher donor density (3.1 × 10^21^ cm^−3^) than nr-Fe_2_O_3_ (1.3 × 10^20^ cm^−3^), along with a five times smaller depletion width of approximately 1.3 nm, compared to 6.4 nm for nr-Fe_2_O_3_ (Fig. 3b). A higher donor density indicates that tp-Fe_2_O_3_ is more conductive due to its more uniform Ti doping distribution. A smaller depletion layer width in tp-Fe_2_O_3_ indicates enhanced charge accumulation near the interface, which not only minimizes bulk recombination losses but also improves charge extraction efficiency at the interface [61]. These features directly address the intrinsically short hole diffusion length of hematite [62].

We further performed electrochemical impedance spectroscopy (EIS) for both tp-Fe_2_O_3_ and nr-Fe_2_O_3_ photoanodes under illumination to evaluate the charge recombination process. The EIS-derived Bode plots (Fig. 3c) show that the phase peak maxima (*F*_max_) for tp-Fe_2_O_3_ and nr-Fe_2_O_3_ occur at 20.4 and 51.4 Hz, and the corresponding electron lifetimes (*τ*_n_) calculated by the equation, τ_n_ = 1/(2πF_max_), are 7.8 and 3.1 ms, respectively. This result suggests that tp-Fe_2_O_3_ has slower interfacial recombination and more efficient separation compared to those of nr-Fe_2_O_3_. In addition, potentiostatic electrochemical impedance spectroscopy (PEIS) measurements in Figs. S23 and S24 show that tp-Fe_2_O_3_ exhibits consistently lower bulk charge transport (*R*_bulk_) and interfacial charge transfer resistance (*R*_ct_), and higher *C*_bulk_ and *C*_trap_, suggesting greater charge accumulation in both the space charge region and surface states [63]. The charge transfer rate constant (*K*_transfer_), calculated as *K*_transfer_ = 1/(*R*_ct_ × *C*_trap_), is higher for tp-Fe_2_O_3_ across the entire potential range, confirming its superior charge transfer kinetics at the hematite/electrolyte interface (Fig. 3d). This is further supported by its much slower transient photocurrent decay and superior charge retention compared to nr-Fe_2_O_3_ (Fig. S25).

Figure 3e shows Kelvin probe force microscopy (KPFM) maps of the surface potential for tp-Fe_2_O_3_ and nr-Fe_2_O_3_ in the dark (left) and under illumination (right) [64]. In these maps, warmer colors (red/yellow) indicate higher surface potential. A shift to warmer colors upon illumination reflects light-induced charge separation and the accumulation of photogenerated carriers at the surface. Compared to the nr-Fe_2_O_3_, the tp-Fe_2_O_3_ photoanode exhibits a more substantial color shift and a larger potential change between light and dark (*V*_light_ − *V*_dark_ = 89 mV; Figs. 3f and S26), indicating more efficient surface charge separation and accumulation at the electrode surface.

Finally, the charge transport (η_transport_) and charge transfer efficiencies (η_transfer_) were calculated using the hole scavenger method (Figs. 3g, h, and S27) [65]. Across the entire potential range, the tp-Fe_2_O_3_ photoanode exhibits consistently higher η_transport_​ and η_transfer_ than those of nr-Fe_2_O_3_. The larger η_transport_​ for tp-Fe_2_O_3_ comes from the suppressed bulk recombination and improved carrier mobility in tp-Fe_2_O_3_, attributed to its pronounced (110) texturing (Figs. 2c and S15) and uniform dopant distribution (Figs. 1e and S16) [48]. The larger η_transfer_ stems from more efficient utilization of interfacial holes, enabled by its hierarchically porous architecture and thin lateral grain size (Fig. 2e), which promotes effective charge separation and accelerates surface reaction kinetics (Fig. 3d-f) [43]. Notably, while high transfer efficiencies (over 80%) are occasionally reported, simultaneously achieving a high transport efficiency exceeding 30% is particularly significant for hematite photoanodes, distinguishing tp-Fe_2_O_3_ from many commonly utilized 1D nanorod structures and even multi-elements-doped systems.

Activation energies (E_a_) for the OER were determined by analyzing the Arrhenius plot of temperature-dependent J–V data. J–V curves measured at various temperatures (Fig. S28) were used to construct ln(J) versus 1/T plots (Fig. 3i), and E_a_​ values were extracted from the slopes of the linear fit [66]. The tp-Fe_2_O_3_ exhibits much lower E_a_ (9.9 meV) than nr-Fe_2_O_3_ (27.4 meV), indicating faster kinetics, enhanced charge transfer dynamics, and a reduced energy barrier for water oxidation, consistent with the above charge dynamic analysis results.

### Photoelectrochemical (PEC) Performance

**Fig. 4:**
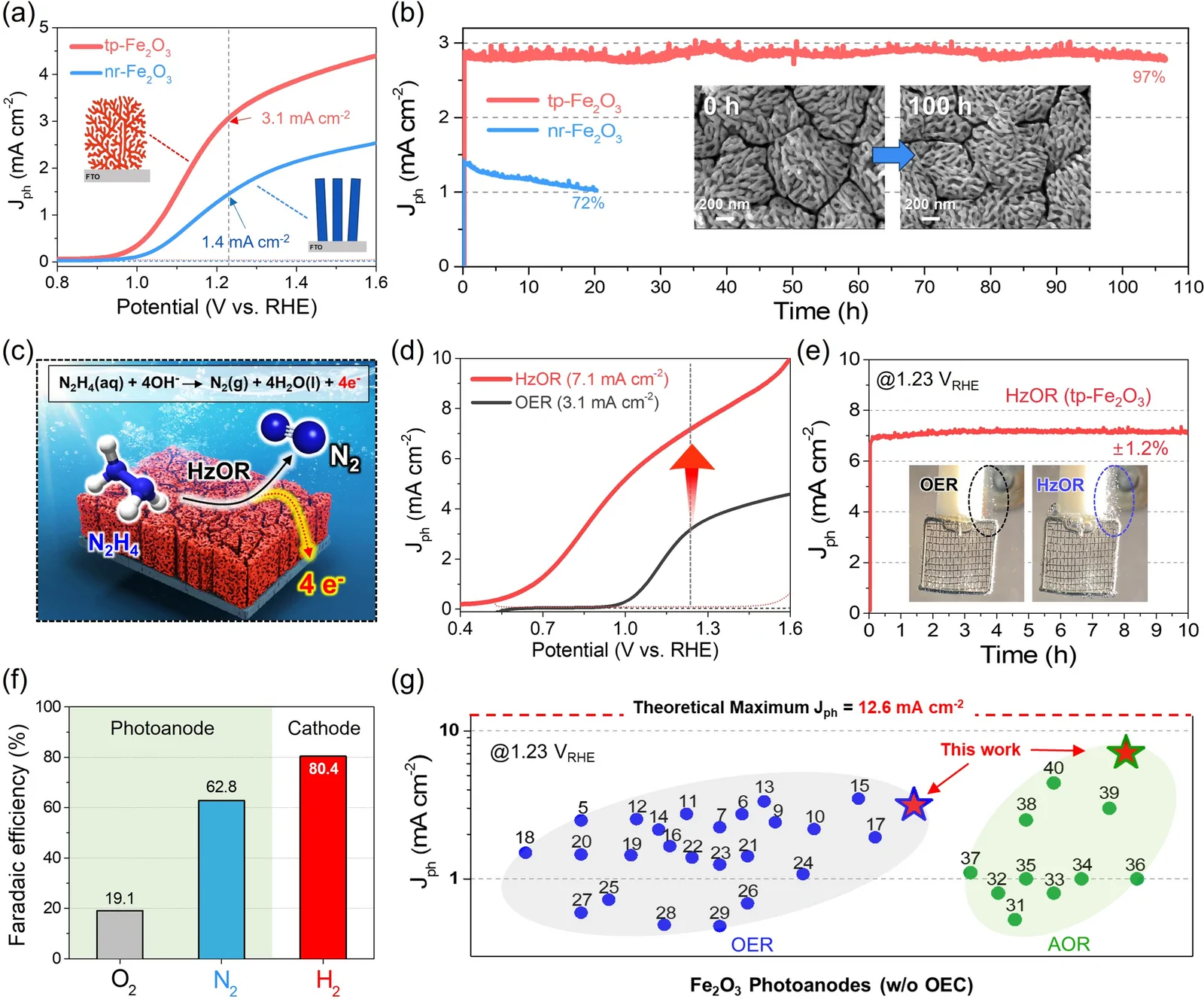
Photoelectrochemical (PEC) oxygen evolution reaction (OER) and hydrazine oxidation reaction (HzOR) performance of Fe_2_O_3_ photoanodes. **a** Photocurrent density-potential (J–V) and **b** photocurrent stability curves of nr- and tp-Fe_2_O_3_ photoanodes in 1 M NaOH (pH 13.6). Inset: SEM morphology change after 100 h of operation. **c** Schematic of PEC HzOR system. HzOR in alkaline electrolyte generates N_2_, H_2_O, and four electrons. **d** J–V and **e** chronoamperometry curves of tp-Fe_2_O_3_ for PEC HzOR. Inset: photographs of H_2_ bubble generation at the Pt cathode. **f** Faradaic efficiency (FE) for O_2_, N_2,_ and H_2_. **g** Literature comparison of Fe_2_O_3_-based photoanodes (without OEC) for OER and various alternative oxidation reactions (AOR), the corresponding reference numbers are taken from Tables S3 and S4

We next investigate how the structural advantages and enhanced charge dynamics of tp-Fe_2_O_3_ affect its PEC performance compared to nr-Fe_2_O_3_ (Fig. 4). Under 1 Sun illumination, tp-Fe_2_O_3_ achieves an OER photocurrent density of 3.1 mA cm^−2^ at 1.23 V_RHE_ (Fig. 4a), more than twice that of nr-Fe_2_O_3_ (1.4 mA cm^−2^). In addition, tp-Fe_2_O_3_ exhibits a higher incident photon-to-current efficiency (IPCE), reaching a peak value of 52% at 390 nm (Fig. S29), along with improved applied bias photon-to-current efficiency (ABPE) and absorbed photon-to-current efficiency (APCE) compared to nr-Fe_2_O_3_ (Fig. S30). As shown in Fig. 4b, tp-Fe_2_O_3_ also exhibits excellent stability, retaining 97% of its initial photocurrent density after 105 h with no apparent morphological change (SEM insets). In contrast, nr-Fe_2_O_3_ retains only 72% after 20 h. In addition, the gas evolution of tp-Fe_2_O_3_ during PEC water splitting was quantified using gas chromatography, and the average Faradaic efficiencies (FE) for H_2_ and O_2_ production were calculated to be approximately 80% (Fig. S31).

Next, we investigated the potential to replace the sluggish OER with the kinetically more favorable HzOR by utilizing tp-Fe_2_O_3_ to enhance H_2_ production [67]. HzOR is appealing because N_2_H_4_ is a widely used chemical compound (Fig. S32) across diverse industries. However, it is highly toxic to health and the environment (Table S1). PEC HzOR coupled with HER provides a strategy for simultaneously remediating N_2_H_4_ to benign N_2_ and boosting H_2_ production (Fig. 4c and Table S2) [68]. HzOR, compared to the OER, proceeds at a substantially lower theoretical potential (− 0.33 V_RHE_) and exhibits superior reaction kinetics [69]. Density functional theory (DFT) free energy profile reveals that the potential-determining step (PDS) for OER on Ti-doped Fe_2_O_3_ (110) is *OH to *O with an energy barrier 1.26 eV, whereas for the HzOR, the *N_2_H_4_ to *N_2_H_3_ PDS step exhibits a much lower energy barrier of 0.33 eV. This value is even smaller than that of the PDS for H_2_O_2_ oxidation (0.48 eV), indicating much more favorable thermodynamics for HzOR (Figs. S33 and S34).

Figure 4d shows that the photocurrent density of the tp-Fe_2_O_3_ photoanode is increased from 3.1 mA cm^−2^ for OER to 7.1 mA cm^−2^ for HzOR at 1.23 V_RHE_. The onset potential is greatly shifted cathodically to 0.6 V_RHE_. Notably, the tp-Fe_2_O_3_ photoanode with HzOR achieves a photocurrent density of approximately 10 mA cm^−2^ at a higher potential of 1.6 V_RHE_, corresponding to nearly 80% of Fe_2_O_3_’s theoretical maximum photocurrent density. Similar benefits of using HzOR over OER were also observed for the nr-Fe_2_O_3_ photoanode (Fig. S35). The tp-Fe_2_O_3_ photoanode also exhibits good stability, with negligible degradation over 10 h of continuous operation under HzOR (Fig. 4e). During the stability test, we observed vigorous H_2_ bubble formation on the Pt mesh counter electrode with HzOR, in contrast to the OER condition, and the H_2_ production rate under HzOR is 2.2 times higher than that driven by OER (Fig. S36). The measured FE were 80.4% for H_2_, 62.8% for N_2_, and 19.1% for O_2_ (Figs. 4f and S37), and the combined amount of anodic gas (N_2_ and O_2_) was approximately half that of the evolved H_2_, consistent with the expected stoichiometry.

Figure 4g compares the photocurrent densities at 1.23 V_RHE_ for Fe_2_O_3_ photoanodes without oxygen evolution co-catalysts (OEC) under both OER and AOR conditions. In both cases, our tp-Fe_2_O_3_ photoanode surpasses most of the previously reported values for bare Fe_2_O_3_ systems. To the best of our knowledge, our tp-Fe_2_O_3_ achieves a high photocurrent density of 7.1 mA cm^−2^ at 1.23 V_RHE_ under HzOR conditions, which exceeds all previously reported values for Fe_2_O_3_-based photoanodes, under both water oxidation and alternative anodic reaction pathways (Tables S3 and S4).

### Solar-Powered Hydrazine Oxidation Reaction Coupled with H_2_ Production

**Fig. 5:**
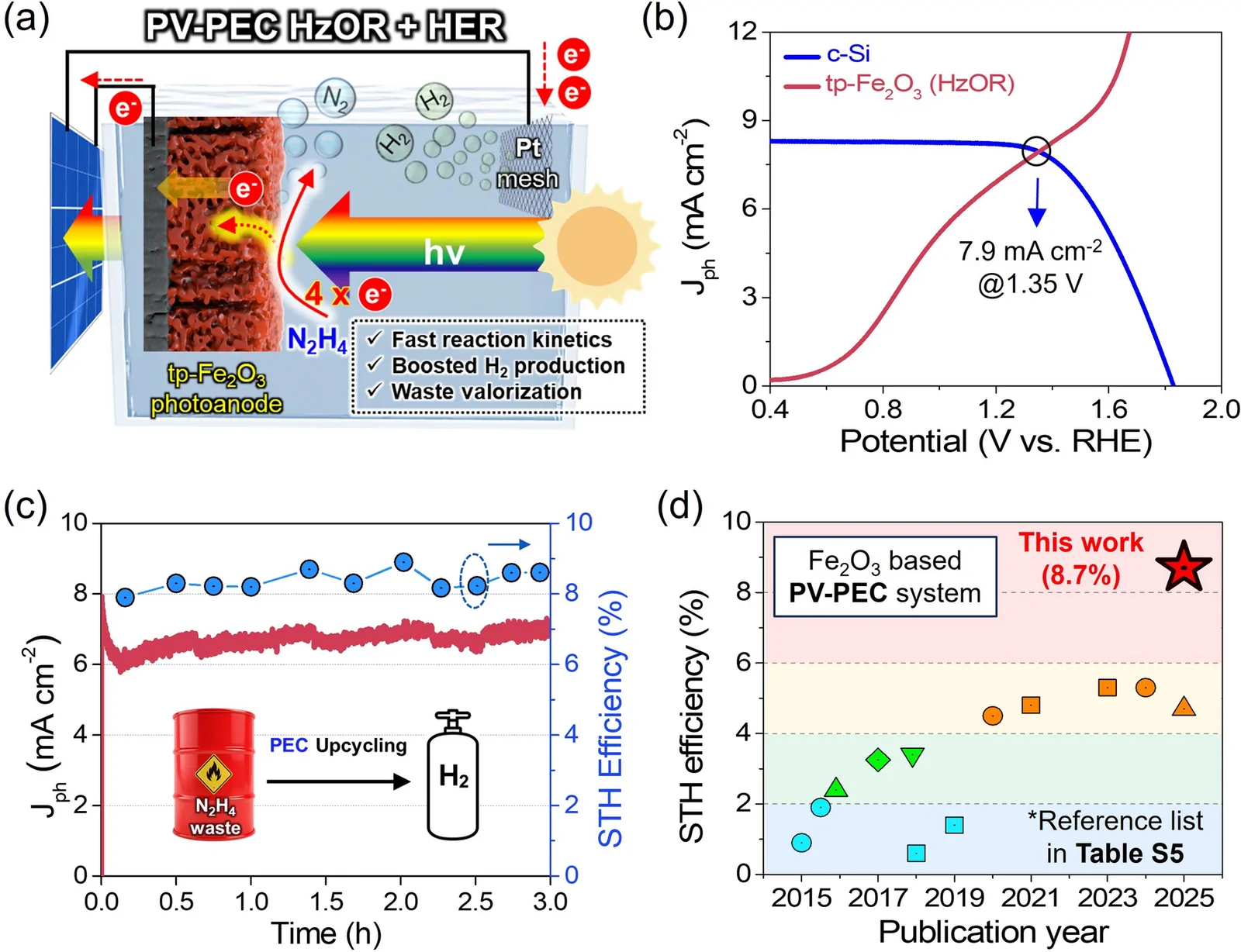
Integrated PV-PEC system for hydrazine-boostered hydrogen production without external bias. **a** Schematic illustration of the integrated PV-PEC tandem system. **b** J–V curves of the c-Si solar cell (behind tp-Fe_2_O_3_ photoanode) and PEC HzOR cell under simulated sunlight illumination (1 sun, 100 mW cm^−2^). The intersection indicates the operating point for unbiased water splitting. **c** Chronoamperometric stability under bias-free PEC HzOR. **d** Comparison of the solar-to-hydrogen (STH) efficiencies reported for Fe_2_O_3_-based PV-PEC tandem systems

We finally demonstrated the practical applicability of the tp-Fe_2_O_3_ photoanode for solar-powered H_2_ production by integrating a commercial Si solar cell with the tp-Fe_2_O_3_ photoelectrode under HzOR conditions (Figs. 5a and S38). The J–V curves of the Si solar cell (stack behind tp-Fe_2_O_3_) and the tp-Fe_2_O_3_ photoanode under HzOR conditions intersect at 1.35 V and 7.9 mA cm^−2^ (Fig. 5b), confirming the feasibility of spontaneous H_2_ production without external bias. The integrated PV-PEC system enables hydrazine-boosted H_2_ production under continuous solar illumination for 3 h (Fig. 5c), reaching a maximum STH efficiency of 8.7%. To the best of our knowledge, this represents the highest reported STH efficiency for Fe_2_O_3_-based PV-PEC systems (Fig. 5d and Table S5).

## Conclusion

We addressed intrinsic limitations of hematite as a PEC photoanode, including a short hole diffusion length, low conductivity, and sluggish OER kinetics, through a synergistic co-design of the Fe_2_O_3_ photoanode architecture and surface reaction pathway. We developed a new multi-cycle growth and flame annealing method to synthesize a tp-Fe_2_O_3_ photoanode, featuring a strong (110) texture, uniform Ti distribution, an enlarged surface area, hierarchically porosity, and a thinner lateral grain size. In comparison to typical nr-Fe_2_O_3_, this architecture delivers markedly improved bulk charge transport and interfacial charge transfer, yielding an OER photocurrent of 3.1 mA cm^−2^ at 1.23 V_RHE_ without any co-catalyst and exceptional stability over 105 h. When we further replace the sluggish OER with the HzOR, a photocurrent density of 7.1 mA cm^−2^ is achieved at 1.23 V_RHE_, the highest value reported for Fe_2_O_3_-based photoanodes to date. Furthermore, integration of tp-Fe_2_O_3_ into a bias-free PV-PEC tandem system with a commercial Si solar cell yields a solar-to-hydrogen efficiency of 8.7%, representing the highest reported value for Fe_2_O_3_-based PV-tandem devices. These results provide clear design guidelines for Fe_2_O_3_ photoanodes and highlight HzOR as a sustainable, highly efficient alternative pathway for solar fuel production.

## Supplementary Information

Below is the link to the electronic supplementary material.

Supplementary file1 (DOCX 12637 KB)
