# Supplementary material for: Textured and Hierarchically Porous Hematite Photoanode for Efficient Hydrogen Production via Photoelectrochemical Hydrazine Oxidation
Source: Nanomicro Lett. 2026 Jan 8;18:201. doi: 10.1007/s40820-025-02045-z (PMC12783413; doi:10.1007/s40820-025-02045-z)
Supplement: Supplementary file 1 — Supplementary file1 (DOCX 12637 KB) [file 40820_2025_2045_MOESM1_ESM.docx]

Supporting Information for

**Textured and Hierarchically Porous Hematite Photoanode for Efficient Hydrogen Production via Photoelectrochemical Hydrazine Oxidation**

Runfa Tan ^1,2,#^, Yoo Jae Jeong ^1,3,#^, Hyun Soo Han ^6^, Samadhan Kapse ^1,2^, Seong Sik Shin ^4,5^, Xiaolin Zheng ^6,^* and In Sun Cho ^1,2,^*

^1^ Department of Materials Science & Engineering, Ajou University, Suwon 16499, Republic of Korea

^2^ Department of Energy Systems Research, Ajou University, Suwon 16499, Republic of Korea

^3^ Materials Science and Engineering Program, University of Colorado Boulder, Boulder, CO 80303, United States

^4^ SKKU Advanced Institute of Nanotechnology (SAINT) and Department of Nanoengineering, Sungkyunkwan University, Suwon 16419, Republic of Korea

^5^ Department of Energy Science, Sungkyunkwan University, Suwon 16419, Republic of Korea

^6^ Department of Mechanical Engineering, Stanford University, California 94305, United States

^#^Runfa Tan and Yoo Jae Jeong contributed equally to this work.

*Corresponding authors. E-mail: xlzheng@stanford.edu (Xiaolin Zheng); insuncho@ajou.ac.kr (In Sun Cho)

**S1 Experimental section**

**S1.1 Chemicals and reagents**

All chemicals were used as received without further purification. Iron(III) chloride hexahydrate (FeCl_3_·6H_2_O, >98%), urea (>98%), and titanium(III) chloride solution (TiCl_3_, 20.0%) were purchased from Sigma-Aldrich (USA) and Kanto Chemical (Japan). Titanium butoxide (Ti(OBu)_4_, 97%) and 2-methoxyethanol (99.3%) were obtained from Sigma-Aldrich and Alfa Aesar, respectively. Hydrazine monohydrate (N_2_H_4_·H_2_O, 98%) was purchased from Sigma-Aldrich and handled with caution due to its toxicity. Deionized (DI) water with a resistivity of 18.2 MΩ·cm was used in all solution preparations. Fluorine-doped tin oxide (FTO) glass substrates (sheet resistance ~15 Ω sq^-1^, Pilkington) were sequentially cleaned via ultrasonication in acetone, isopropanol, and DI water for 15 min each, followed by nitrogen drying.

**S1.2 Physicochemical characterization of the photoanode**

The texture coefficient (TC) was calculated from the XRD patterns using the following equation:

${TC}_{(hkl)}=\frac{I(hkl)}{\sum I(hkl)}\frac{I^{^{\circ}}(hkl)}{\sum I^{^{\circ}}(hkl)}$,

where $I$ is the measured XRD peak intensity, $I_{0}$ is the reference XRD intensity from JCPDS #33-0664 (Fe_2_O_3_).

Lateral grain size is the characteristic width of an individual crystallographic grain measured perpendicular to the material’s principal (growth/elongation) axis. In this work, the quantification was based on SEM/TEM images.

The work of adhesion was calculated from the contact angles using the Young-Dupré equation:$W_{adhesion}=\gamma L(1+\cos\left( \theta\right))$, where $W_{adh}$ is the work of adhesion (interfacial adhesion energy), $\gamma L$ is surface tension of the water (72.8 mJ m^-2^ at room temperature), is the contact angle (degrees).


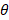


**S1.3 PEC performance measurements and efficiency assessment**

Photoelectrochemical (PEC) measurements were conducted in a three-electrode configuration using a Biologic SP-200 potentiostat. The Fe_2_O_3_ photoanode served as the working electrode, while a platinum wire and an Ag/AgCl (3M KCl) electrode served as the counter and reference electrodes, respectively. The light source was a 150 W Xe lamp equipped with an AM 1.5G filter (solar simulator, LCS-100, Newport, 100 mW cm^-2^).

Transient photocurrent responses were used to assess charge separation and recombination kinetics. Mott–Schottky measurements were performed in the dark at a frequency of 1 kHz. The flat band potential (*E_fb_*) and carrier density (*N_d_*) were calculated according to the following equation:

$\frac{1}{C^{2}}=\frac{2}{e\varepsilon\varepsilon_{0}N_{d}}\left[ \left( E-E_{fb} \right)-\frac{\mathrm{kT}}{e} \right]$,

where *C* is the space charge capacitance (F cm^–2^), e is the electron charge (1.6 × 10^−19^ C), ε and ε_0_ represent the vacuum permittivity (8.85 × 10^–12^ F m^–1^) and the relative dielectric constant of hematite (80), *E* is the electrode applied potential, k is the Boltzmann’s constant (1.38 × 10^–23^ J K^–1^) and T is the absolute temperature (K). The depletion layer width (W_D_) was calculated according to the following equation:

$w_{D}$ = $\sqrt{\frac{2\varepsilon_{r}\varepsilon_{0}\left( V_{\mathrm{bi}}-V \right)}{qN_{D}}}$ ,

where$\varepsilon_{r}$ is the relative permittivity (dielectric constant) of hematite (80), $\varepsilon_{0}$​ is the permittivity of free space (8.85×10^−14^F/cm), $V_{bi}$is the built-in potential (0.6 V), $V$ is the applied bias voltage (For simplicity, assuming no external bias for depletion width calculation), $q$is the elementary charge (1.6×10^−19^C), $N_{D}$is the donor concentration (obtained from Mott-Schottky analysis).

Faradaic efficiency for gas evolution (H_2_, O_2_, and N_2_) was determined using a gas chromatograph (YL6500GC, Young In Chromass) and calculated using the following equation:

$$Faradaic efficiency \left( FE\% \right)=\frac{amount of gas collected in moles}{J_{ph}\times A\times t/n\times e\times N_{A}}$$

where J_ph_ is the photocurrent density (A cm^-2^), A is the area (cm^2^), t is the time (s), e is the elementary charge (1.602 × 10^−19^ C), n=4 for O_2_/N_2_ (2 for H_2_), and N_A_ is Avogadro’s number (6.02 × 10^23^ /mol).

Incident photon-to-current efficiency (IPCE) was measured at 1.23 V_RHE_ using an IPCE equipment (HS Technologies, PE IPCE). The absorbed photon-to-current efficiency (APCE) spectra were calculated using: APCE(λ) = IPCE(λ)/A(λ), where A(λ) denotes the optical absorbance at each wavelength.

The applied bias photon-to-current efficiency (ABPE) was calculated using:

$A\mathrm{BPE}=\frac{J_{ph}\times(1.23 V- V_{bias})}{P_{light}}$,

Where J_ph_​ is the photocurrent density, $V_{\mathrm{bias}}$ is the applied potential vs. RHE, P_light_​ is the incident light power density.

Solar-to-hydrogen (STH) efficiency was calculated using:

$$S\mathrm{TH}\left( \% \right)=\frac{J_{ph}\times1.23 V\times FE\%}{P_{light}}\times100$$

where 1.23 V represents the theoretical minimum voltage required for water splitting, J_ph_​ is the photocurrent density at the operating point (i.e., the intersection of the J–V curves), FE% denotes the faradaic efficiency for hydrogen evolution, and P_light_​ is the incident light power density.

**S1.4 Computational details**

Density functional theory (DFT) calculations were carried out using the Vienna Ab initio Simulation Package (VASP) [S1]. Exchange–correlation effects were treated within the generalized gradient approximation (GGA) employing the Perdew–Burke–Ernzerhof (PBE) functional [S2]. Core–valence interactions were described with projector augmented-wave (PAW) pseudopotentials [S3], and a plane‑wave kinetic‑energy cutoff of 550 eV was applied throughout. Brillouin-zone integrations used a 9 × 9 × 1 Monkhorst–Pack k-point mesh for all slab models [S4]. Electronic occupations near the Fermi level were smoothed using Marzari–Vanderbilt smearing with a width of 0.05 eV. A vacuum spacing of 15 Å was included along the surface normal to avoid spurious interactions between periodic images. Slab models of the (110) surface of hematite (α‑Fe_2_O_3_, space group R-3c) were constructed for the surface calculations. Structural relaxations proceeded until the residual forces were below 0.01 eV Å^-1^ and the total energy change converged to better than 1×10^-3^ eV per atom.

Catalytic performance and thermodynamic barriers were assessed via computed free energy profiles along the reaction pathway. The Gibbs free energy (G) of each intermediate was obtained from: G = E + ZPE – TS – n e U, where E is the DFT total energy, ZPE is the zero-point energy, T is the temperature, S is the entropy, n is the number of electrons transferred, e is the elementary charge, and U is the applied electrode potential [S5]. Zero-point energy and entropic contributions for adsorbed species were assumed to be negligible relative to those of gas-phase molecules at ambient conditions, whereas gas-phase ZPE and entropic values were taken from the chemical database (<https://janaf.nist.gov>).

**Table S1** Potential harm of hydrazine to health and the environment


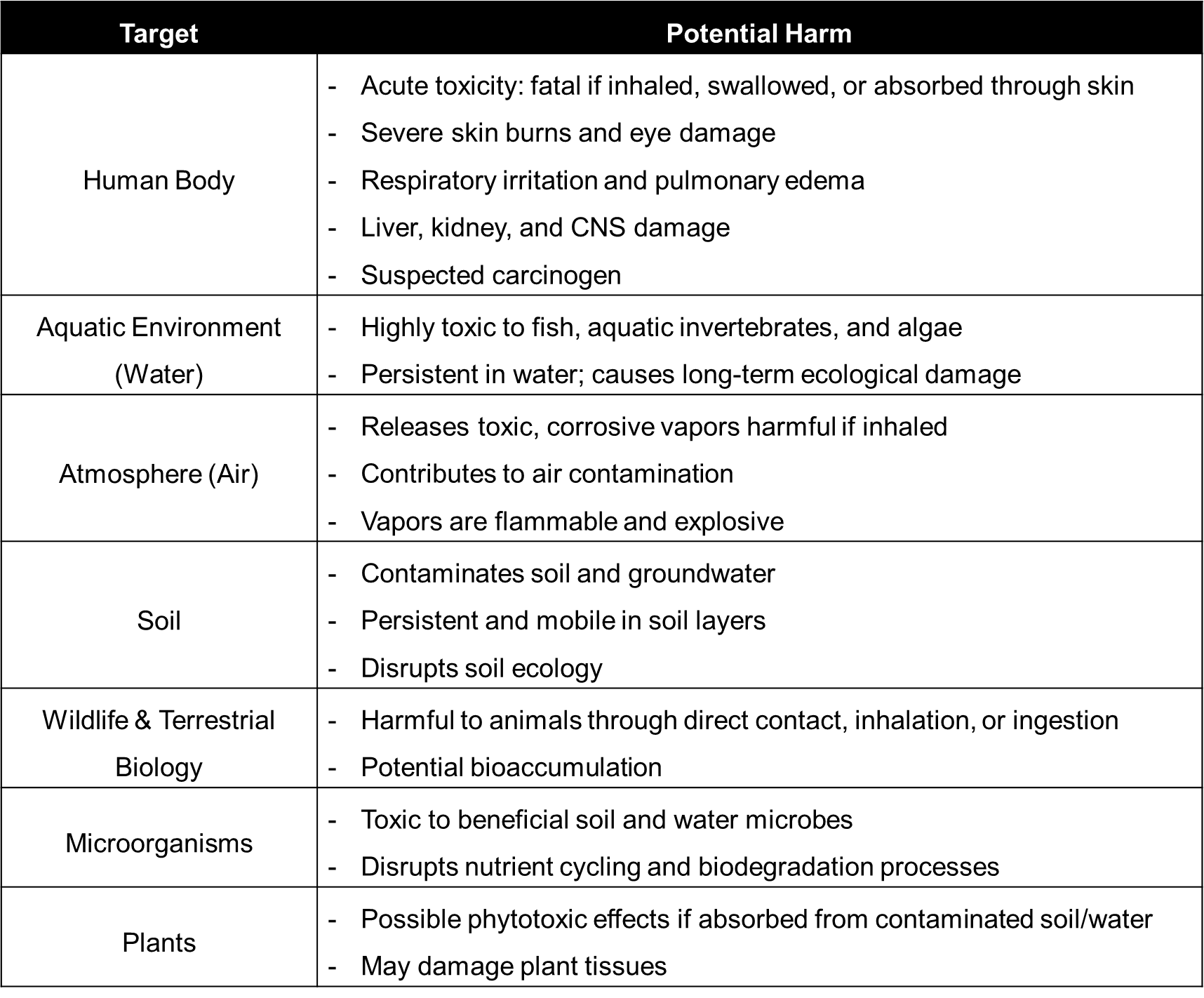


**Table S2** Comparison of hydrazine (N_2_H_4_) wastewater treatment methods: conventional industrial methods vs. photoelectrochemical (PEC) oxidation. PEC oxidation is a cleaner, more controllable, and energy-efficient method


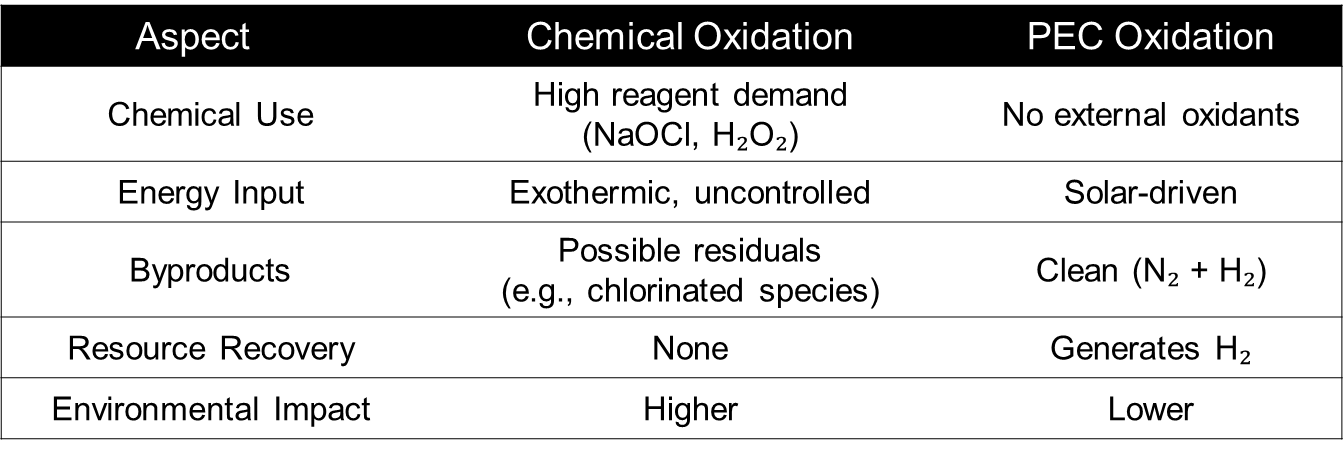


**Discussion:** Industrial effluents often contain hazardous levels of N_2_H_4_, making the effective detoxification and valorization of this compound essential for environmental protection and resource recovery. Conventional hydrazine wastewater treatment typically relies on strong oxidizing agents. However, this approach can produce harmful byproducts and requires significant chemical input and energy, posing environmental and safety concerns. Therefore, developing milder and more sustainable methods for N_2_H_4_ removal is highly desirable. PEC hydrazine oxidation (HzOR), coupled with the hydrogen evolution reaction (HER), provides an efficient strategy for simultaneously remediating N_2_H_4_ and producing H_2_.

**Table S3** Comparison of the various alternative oxidation reaction (AOR) photocurrents over hematite-based photoanodes (at 1.23 V_RHE_). Note that PET denotes poly(ethylene terephthalate); HMF denotes 5-hydroxymethylfurfural


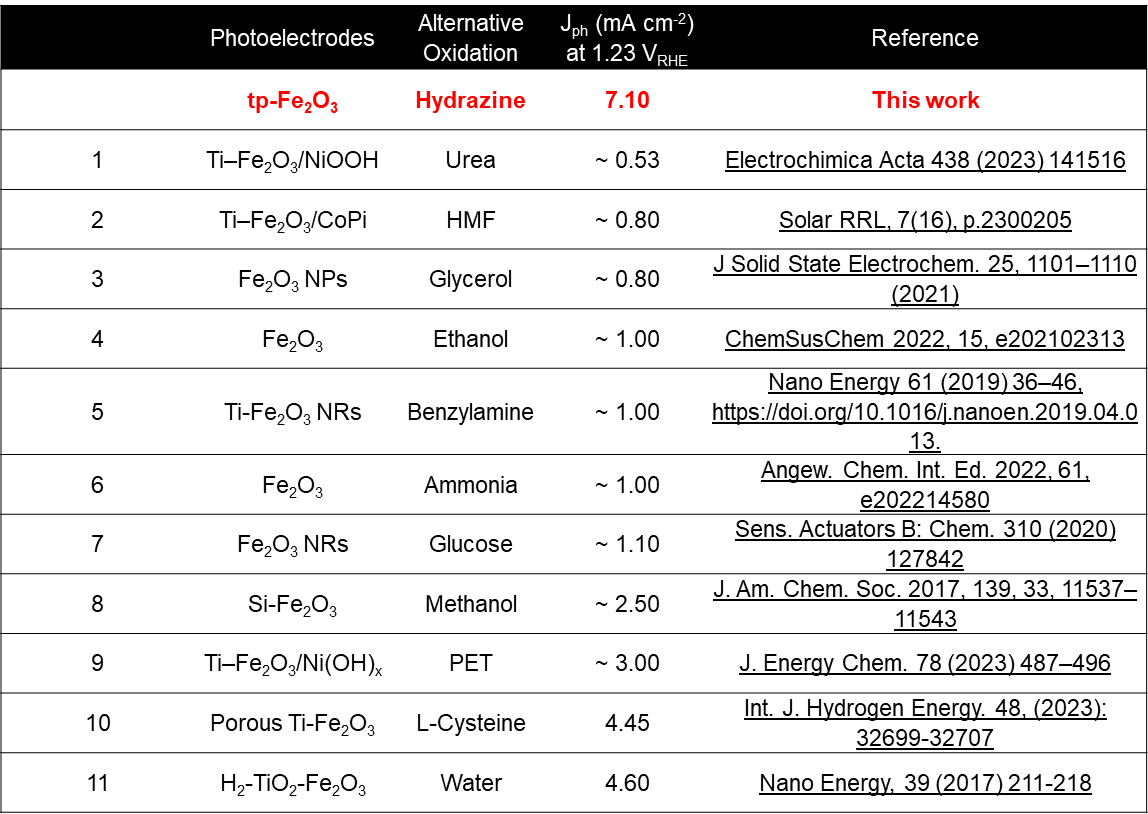


**Table S4** Comparison of the water oxidation photocurrents on hematite-based photoanodes (no oxygen evolution cocatalysts loading at 1.23 V_RHE_)


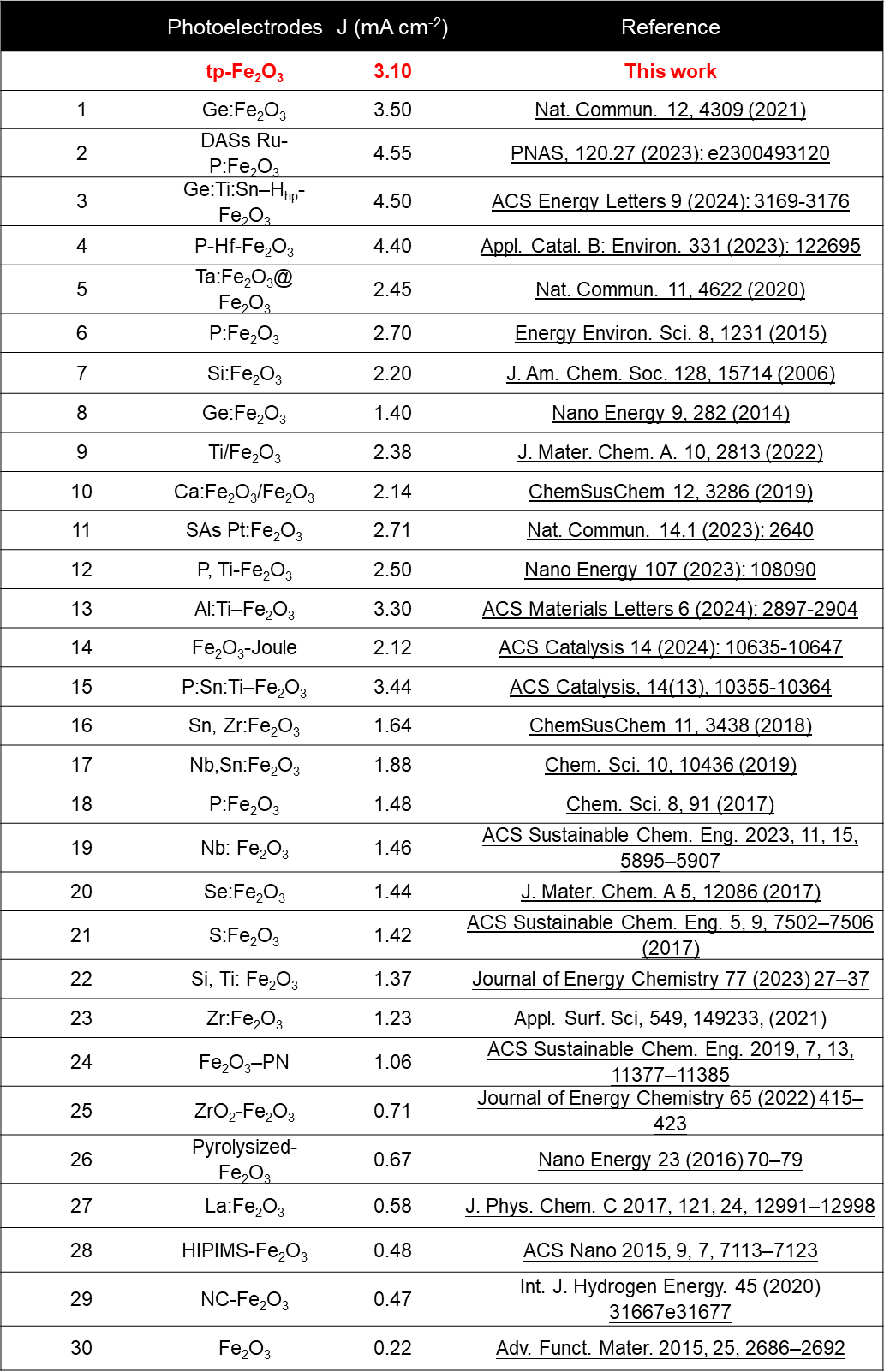


**Table S5** Comparison of the solar-to-hydrogen efficiency (STH) values on hematite-based photoanodes based on a PV-PEC tandem system


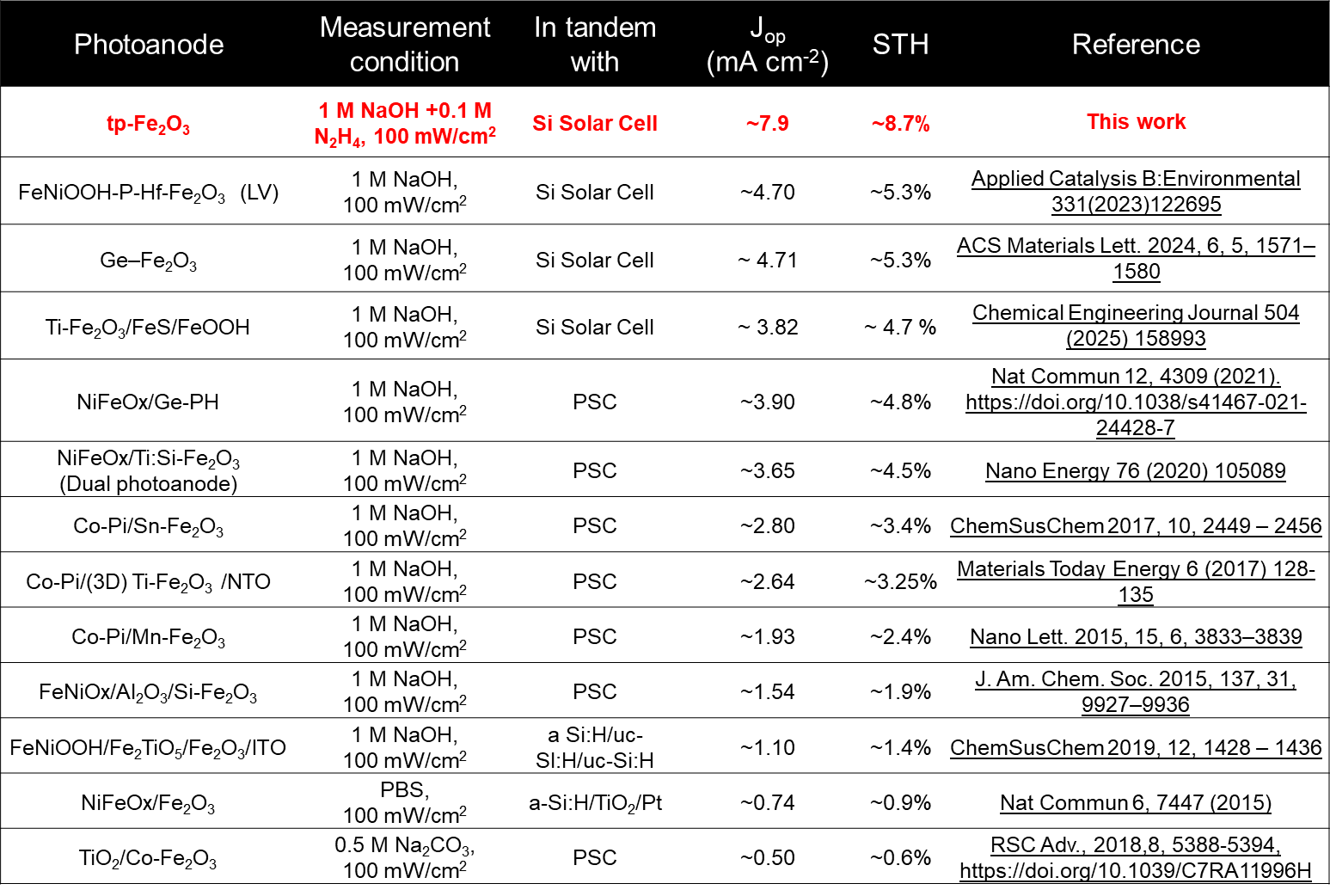


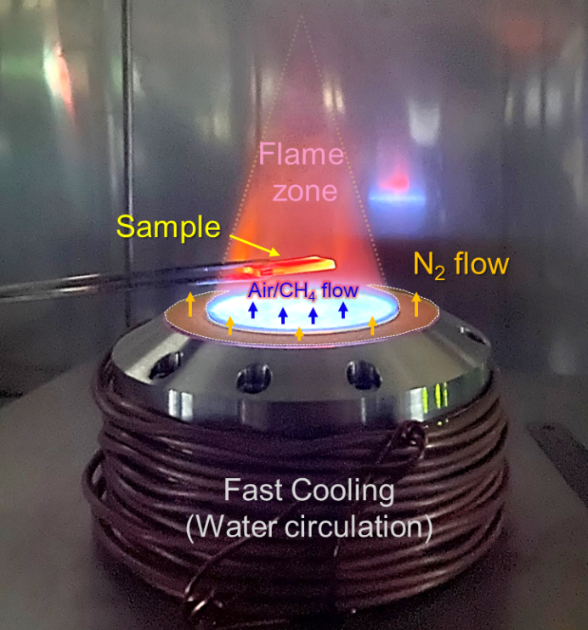


**Fig. S1** Photograph of the flame treatment setup and process. Fuel: CH_4_, Oxidizer: Air. Equivalence ratio (*Φ*) = 0.7. Sample size = 1.5 cm x 2.5 cm

**
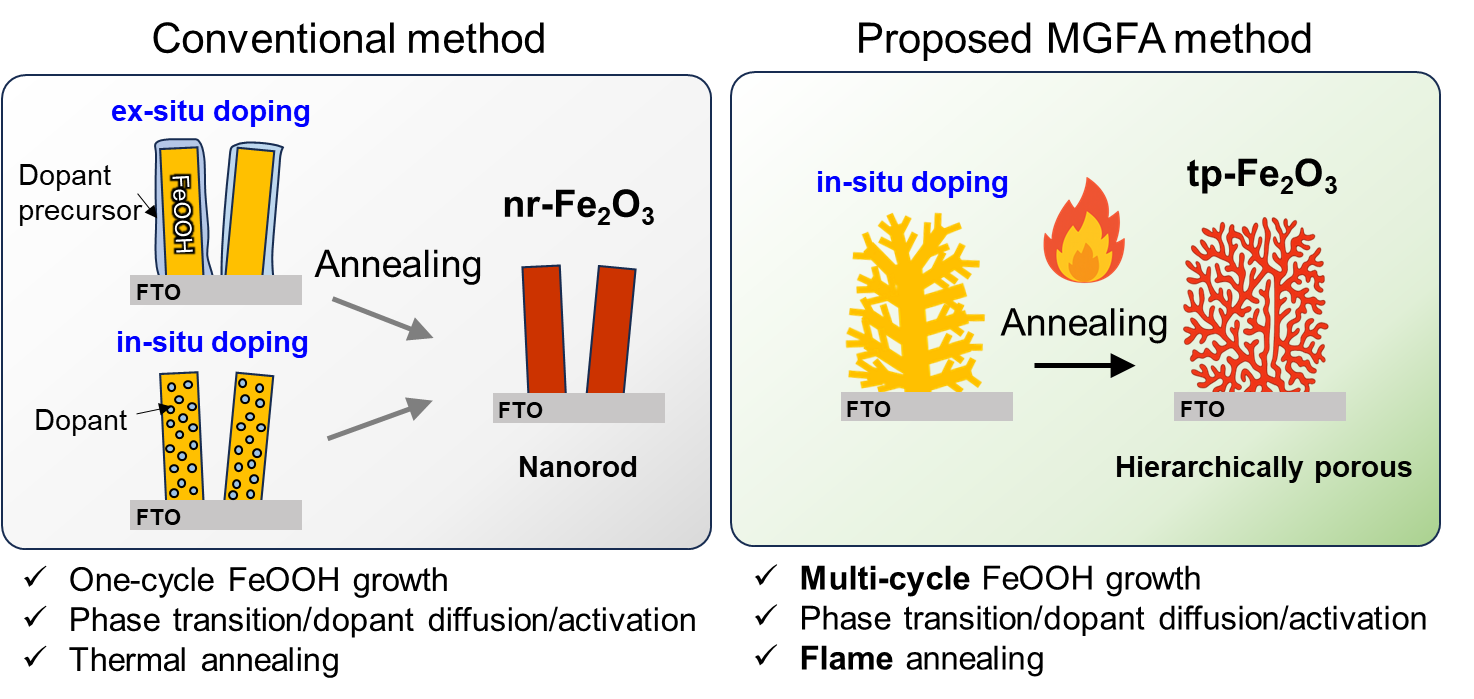
**

**Fig. S2** Conventional method vs. Multi-cycle growth and Flame annealing (MGFA) method for the synthesis and doping of Fe_2_O_3_ photoanode. The multi-cycle solution growth promotes branched morphology, while subsequent rapid flame annealing facilitates uniform dopant incorporation and the formation of a distinctive hierarchical architecture. Additionally, Flame annealing promotes rapid conversion of FeOOH to Fe_2_O_3_ without damaging the FTO substrate


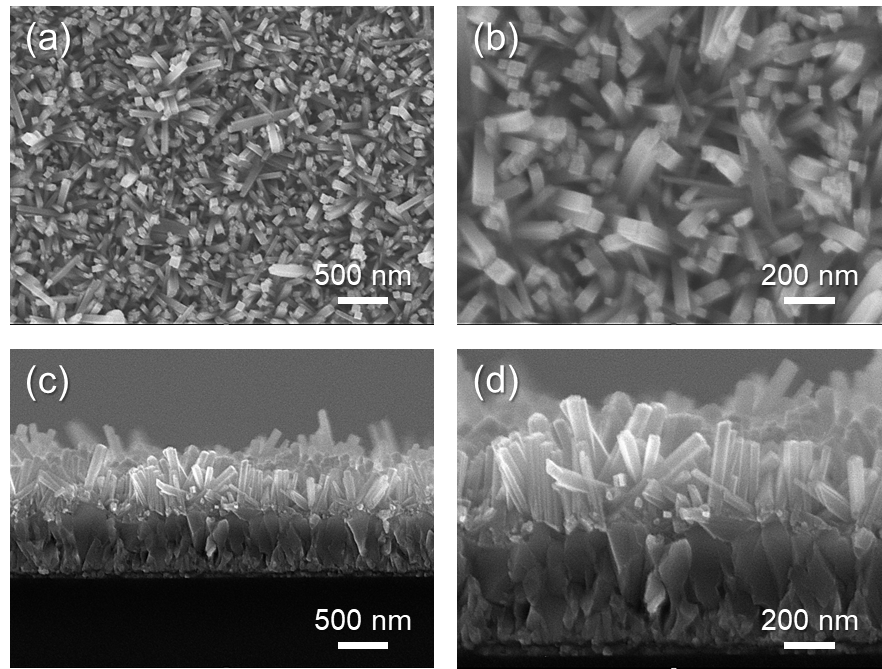


**Fig. S3** SEM images of single-cycle-grown FeOOH nanorods**. a**, **b** Top-view SEM images at low and high magnification showing the uniform growth of vertically aligned FeOOH nanorods. **c**, **d** Corresponding cross-sectional SEM images of the FeOOH nanorods. The single-cycle chemical bath deposition yields well-defined nanorods


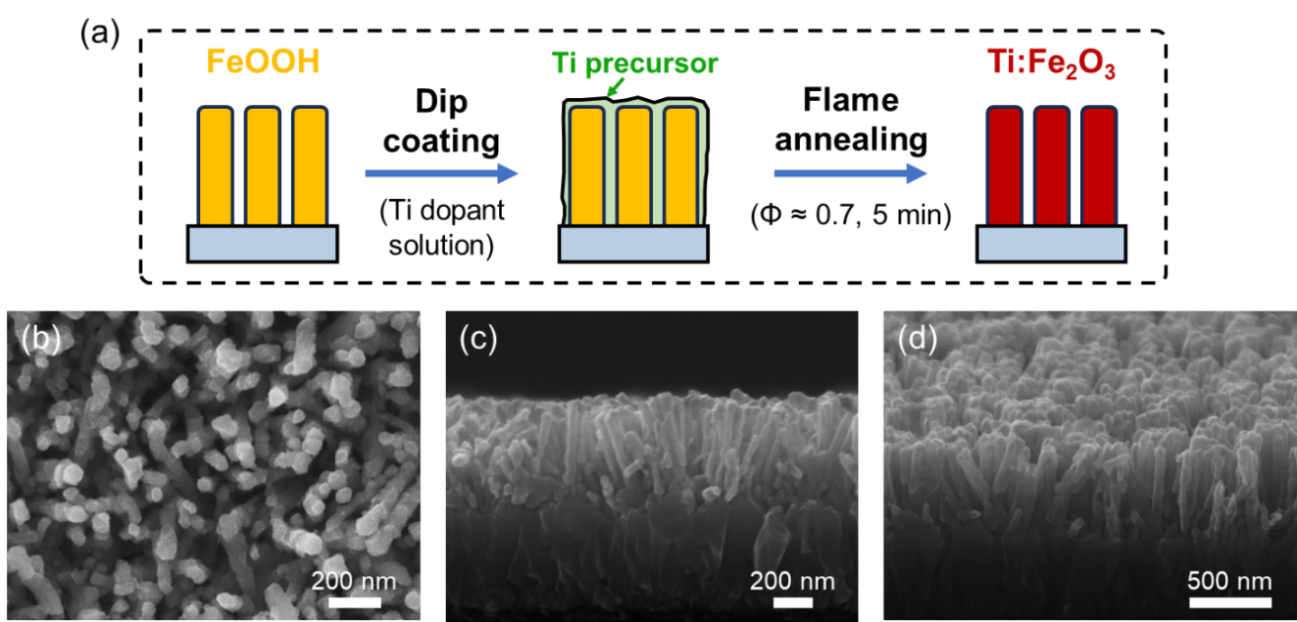


**Fig. S4** nr-Fe_2_O_3_ synthesis method and morphology. **a** Schematic process. SEM images of the synthesized nr-Fe_2_O_3_: **b** top, **c** cross, and **d** tilted view


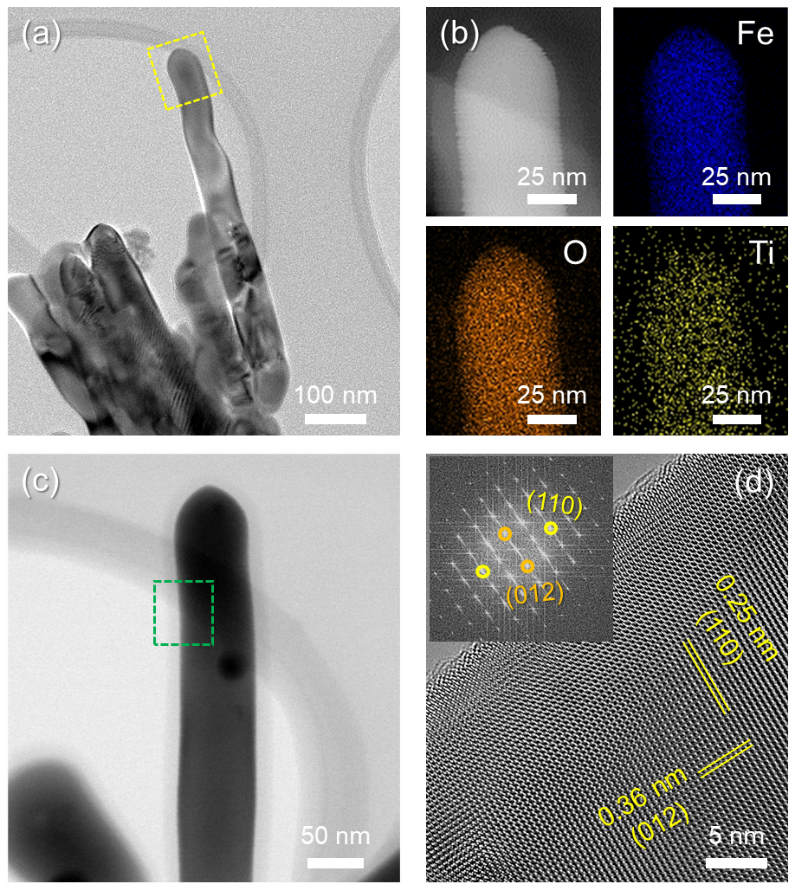


**Fig. S5** TEM analysis of nr-Fe_2_O_3_. **a** Low magnification TEM images. **b** TEM-EDS elemental mapping images. **c** High magnification TEM images. **d** High resolution-TEM and corresponding FFT image. One-dimensional nanorods with high crystallinity and (110) texture was identified


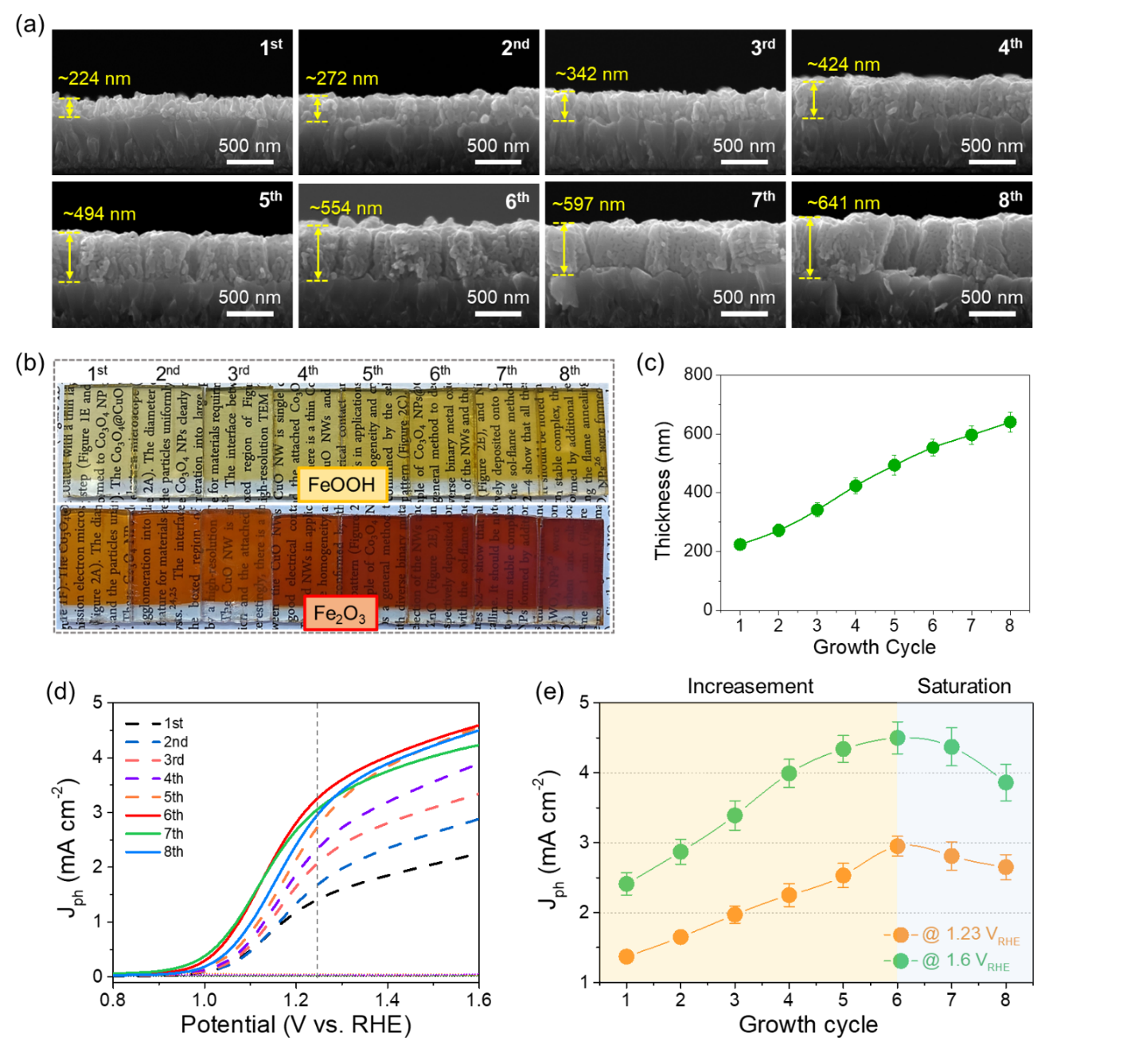


**Fig. S6** Effects of growth cycle on the thickness and J-V curves of tp-Fe_2_O_3_ film. **a** Cross-sectional SEM images of tp-Fe_2_O_3_ with different growth cycles. **b** Photograph of FeOOH and corresponding Fe_2_O_3_ samples. **c** Film thickness vs. growth cycle. **d** Growth cycle-dependent J-V curves. **e** Growth cycle dependent photocurrent density (J_ph_) values at 1.23 V_RHE_ and 1.6 V_RHE_. The 6^th^ cycle sample gives the highest J_ph_ values


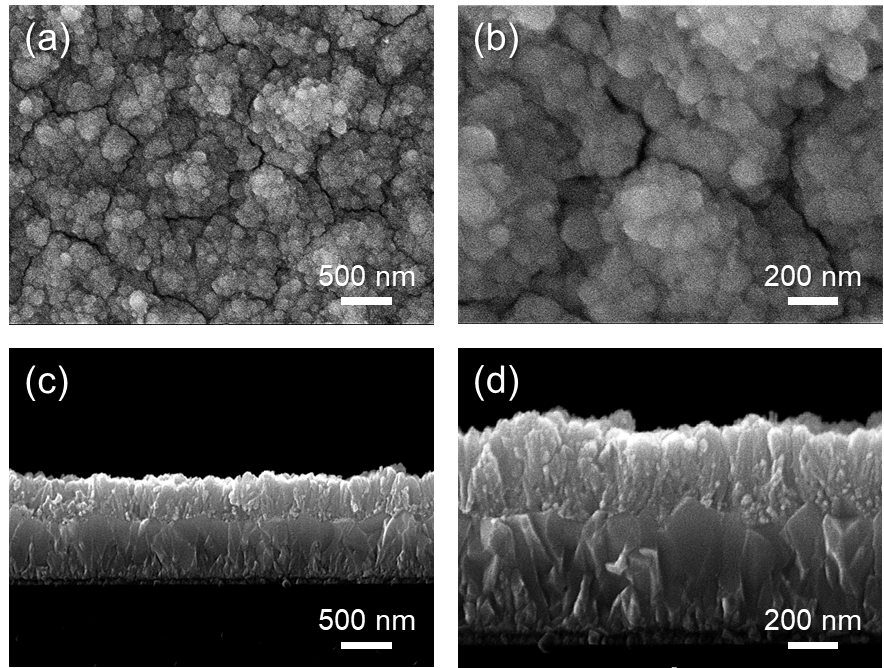


**Fig. S7** SEM images of multi-cycle-grown cauliflower-like branched FeOOH. **a**, **b** Low- and high-magnification top-view SEM images showing the densely packed, cauliflower-shaped morphology of the FeOOH. **c**, **d** Cross-sectional SEM images at low and high magnification, revealing vertically aligned cauliflower-like morphology with extensive secondary branching. The multi-cycle chemical bath deposition process leads to increased structural complexity and higher branch density compared to single-cycle growth


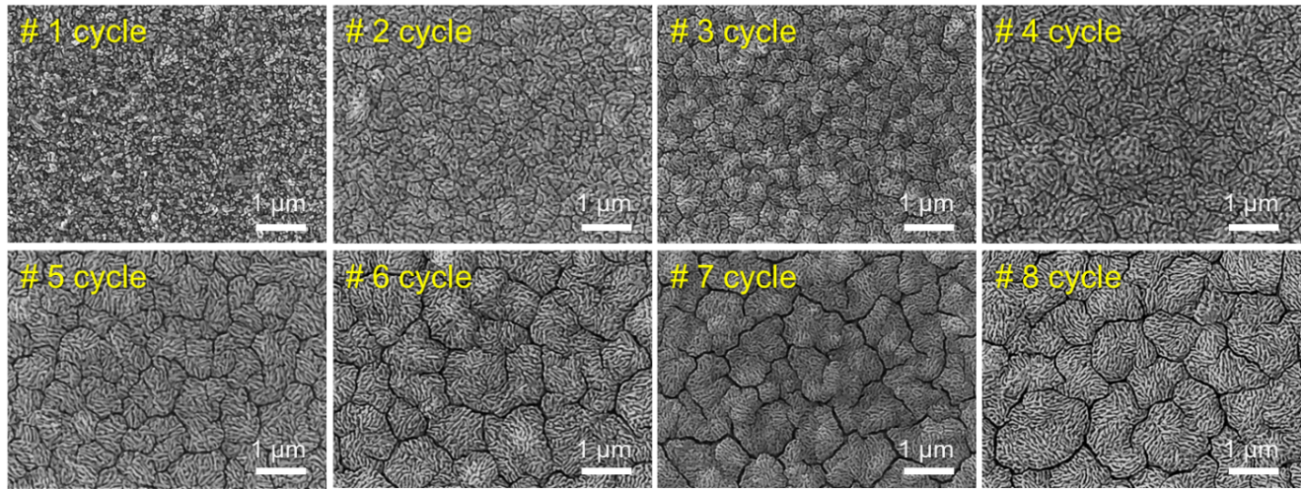


**Fig. S8** Effect of growth cycles on the surface morphology. Low-magnification SEM images of Fe_2_O_3_ films grown with different growth cycles. High surface uniformity is maintained regardless of growth time, with bi-continuous structures becoming more prominent at higher cycle numbers


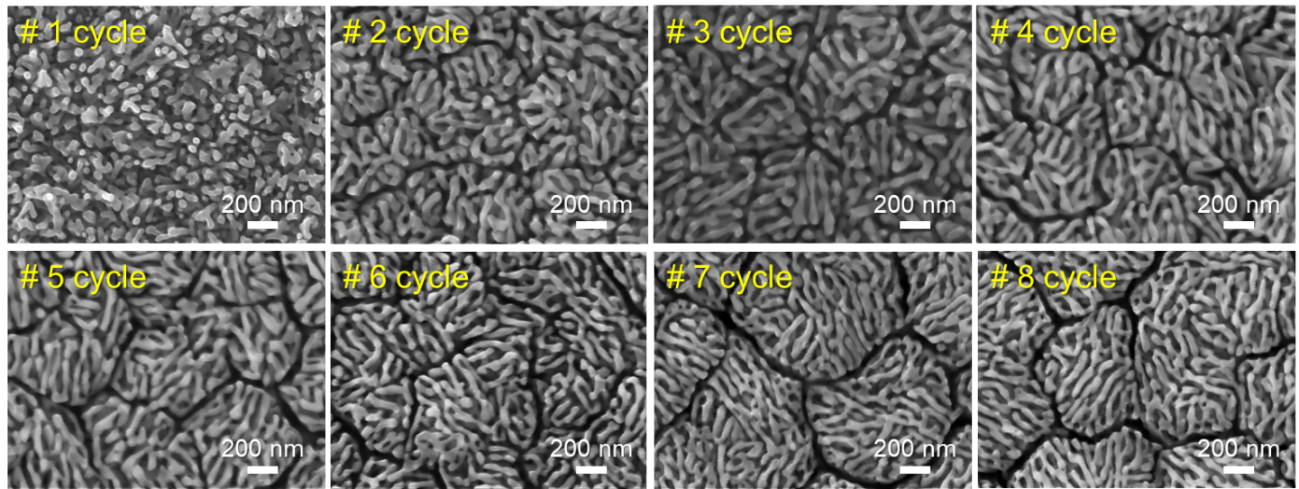


**Fig. S9** Effect of growth cycles on the surface morphology. High-magnification SEM images of Fe_2_O_3_ films with different growth cycles


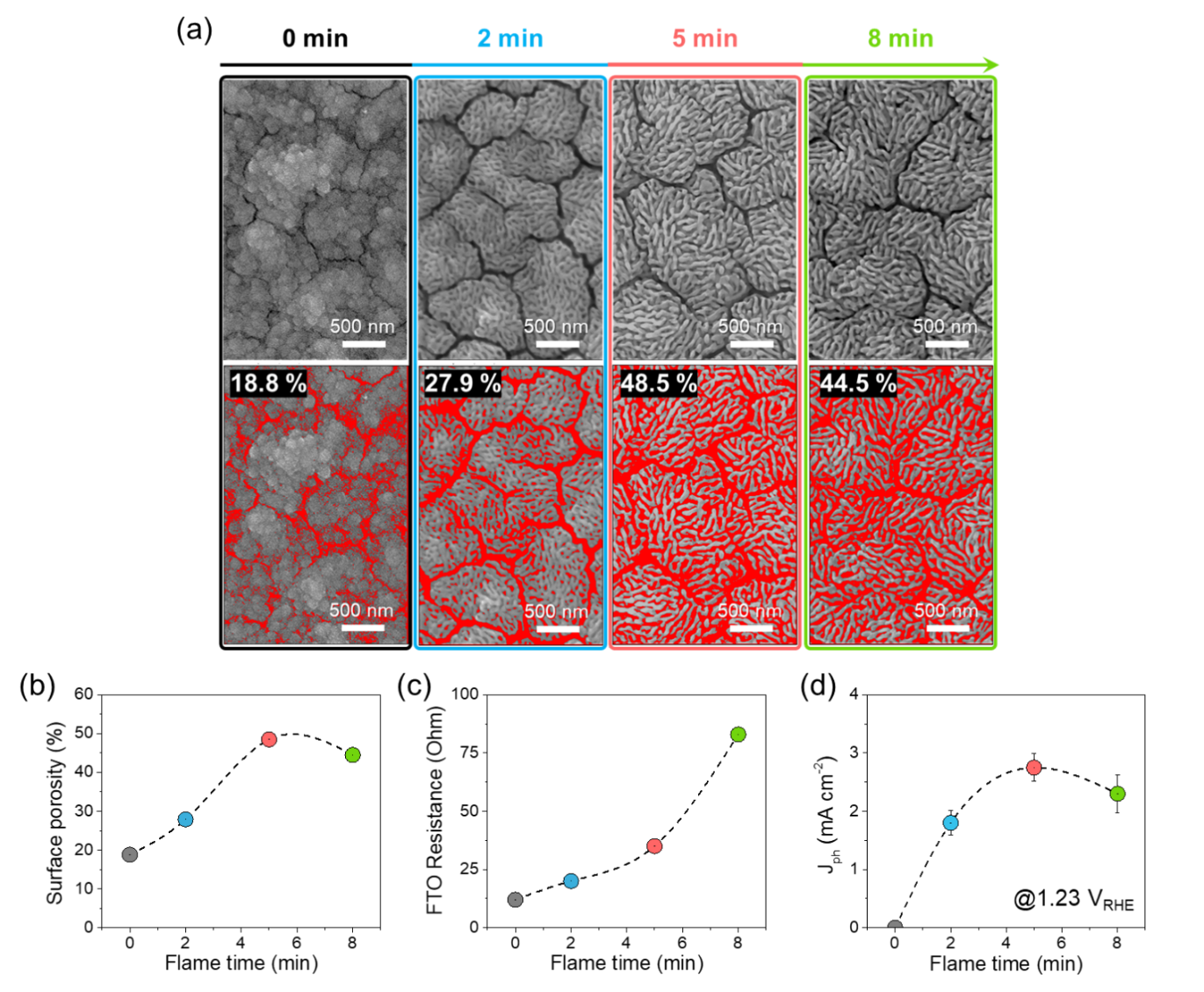


**Fig. S10** Effect of flame annealing duration on surface morphology and porosity. **a** SEM images showing morphological evolution with increasing annealing time, with red-highlighted regions indicating porous areas. **b** Quantified surface porosity fractions obtained by ImageJ analysis. **c** FTO conductivity and **d** photocurrent density (J_ph_) at 1.23 V_RHE_ as a function of flame annealing duration

**Discussion:** With increasing flame treatment time, the porosity of the hematite films gradually evolves. At 0 min, the surface is densely packed with small, irregular grains and shows very low visible porosity, likely due to incomplete crystallization. After 2 min of flame treatment, larger grains with noticeable gaps emerge, indicating the onset of a porous, bio-continuous structure. At 5 min, the porosity reaches its peak, with a well-developed fibrous and interconnected network formed by optimal thermal diffusion. However, by 8 min, the structure becomes more compact due to grain coalescence or sintering, leading to a slight reduction in porosity.


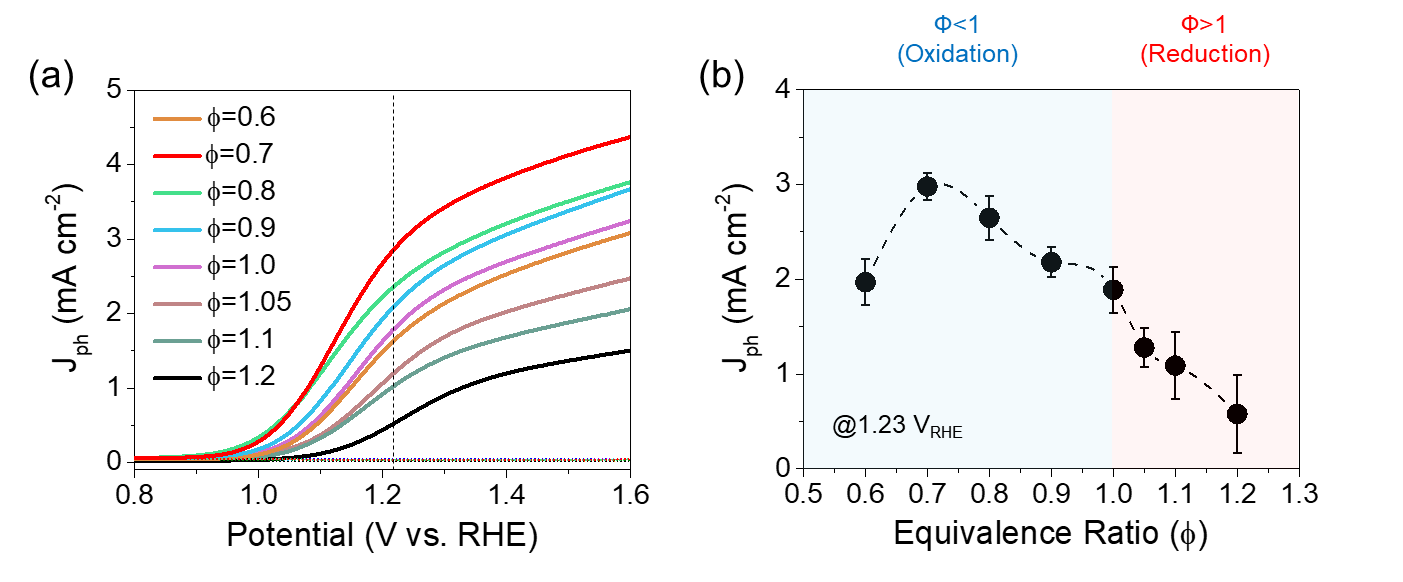


**Fig. S11** Effect of flame atmosphere on the PEC performance of Fe_2_O_3_. **a** J–V curves of tp-Fe_2_O_3_ photoanodes annealed under different flame equivalence ratios (Φ). **b** Corresponding photocurrent densities at 1.23 V_RHE_, showing higher activity under oxidizing flame conditions (Φ < 1)


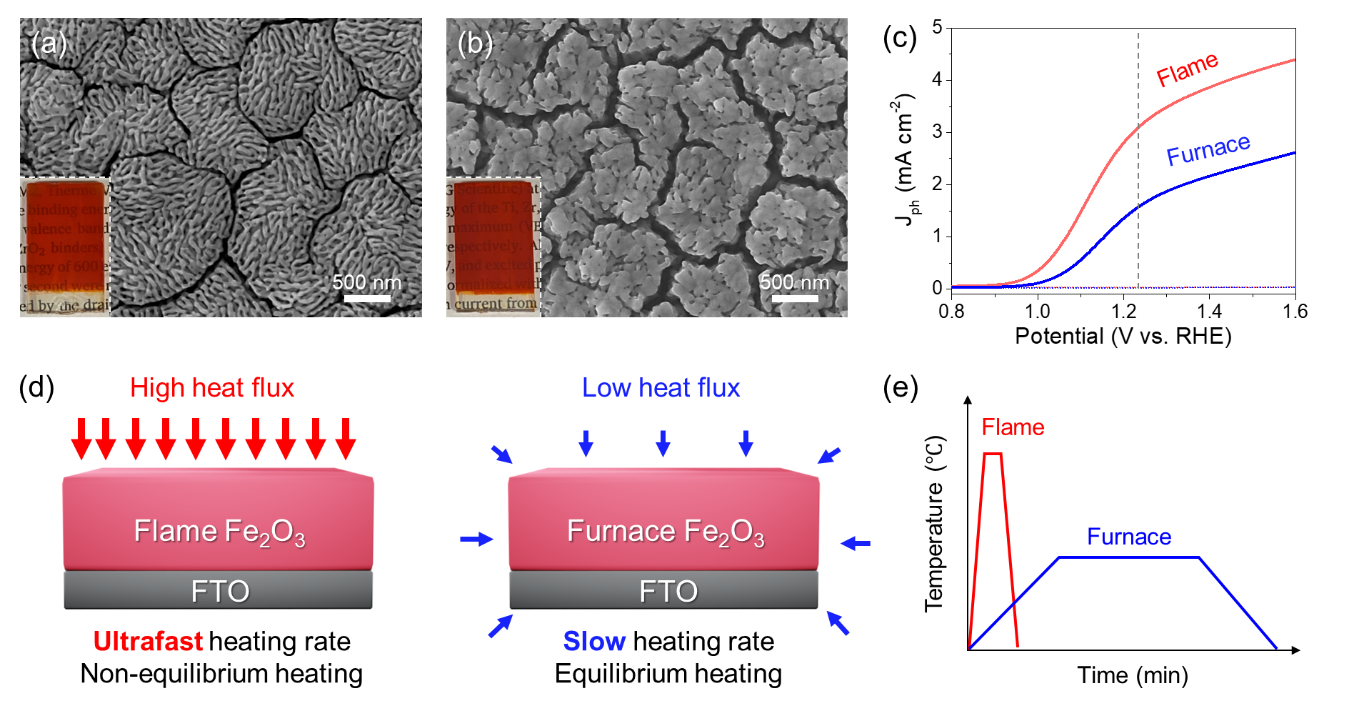


**Fig. S12** Conventional furnace vs. flame annealing: Morphology and J-V curve. SEM image of **a** flame-annealed and **b** furnace-annealed Fe_2_O_3_ photoanodes. **c** J-V curves. **d** Comparison of heat transfer mechanisms between flame and furnace annealing. **e** Temperature profile comparison: flame vs. furnace annealing

**Discussion:** The flame-induced ultrafast phase transformation is crucial for forming a complex, interconnected 3D network structure. The ultrafast heating rate (> 100 ^o^C s^-1^) leads to rapid decomposition/transformation of FeOOH into Fe_2_O_3_ through accelerated dehydration and the release of water vapor, which generates intrinsic porosity. In addition, the anisotropic heat flux and localized thermal stress produced during this process induce micro-scale expansion and contraction, further facilitating the development of hierarchical pores. In contrast, furnace annealing involves a slower temperature increase, enabling uniform heat distribution and gradual FeOOH decomposition. This slow heating promotes grain growth and merging, resulting in a compact, dense morphology. Additionally, the isotropic heat distribution reduces surface stress, thereby limiting the formation of porosity.


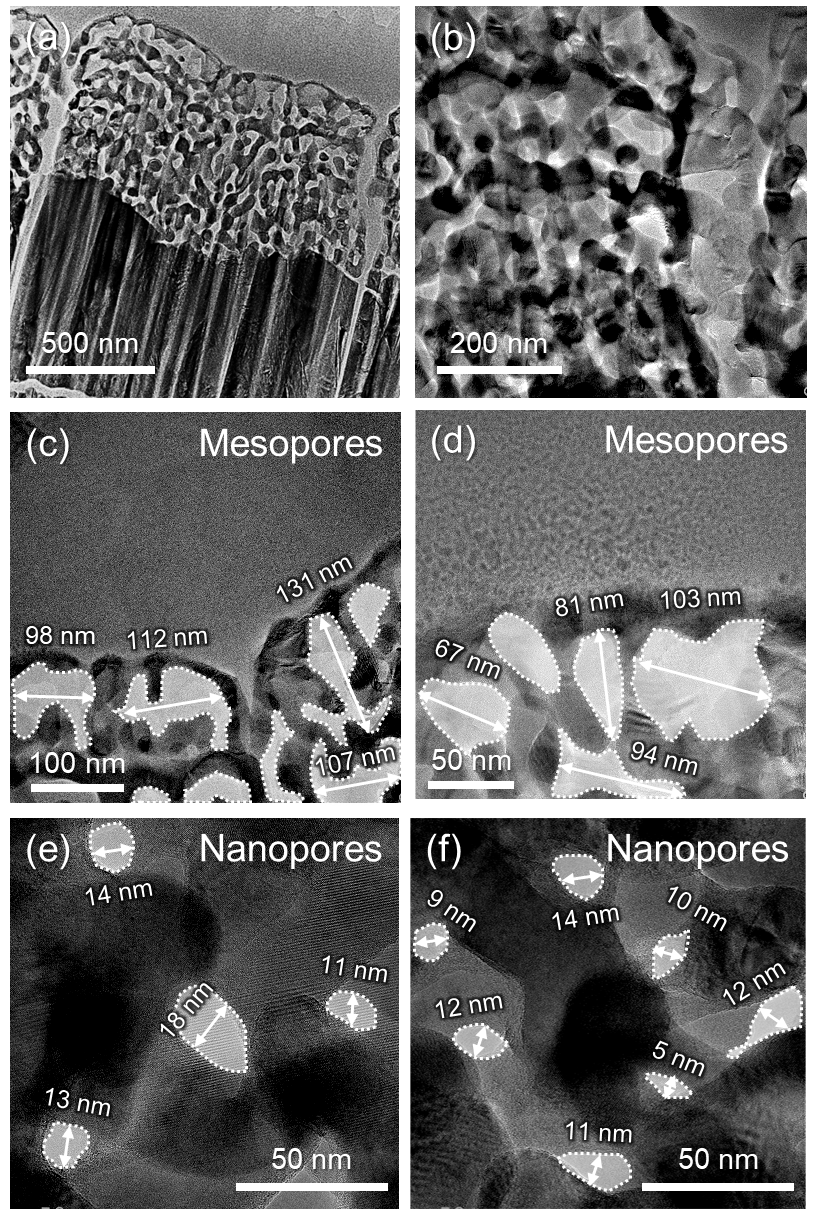


**Fig. S13** Multiscale porosity characterization of tp-Fe_2_O_3_ via TEM analysis. **a**, **b** Low-magnification: interconnected porous network. **c**, **d** Medium-magnification: abundant mesopores. **e**, **f** High-magnification: uniformly distributed nanopores


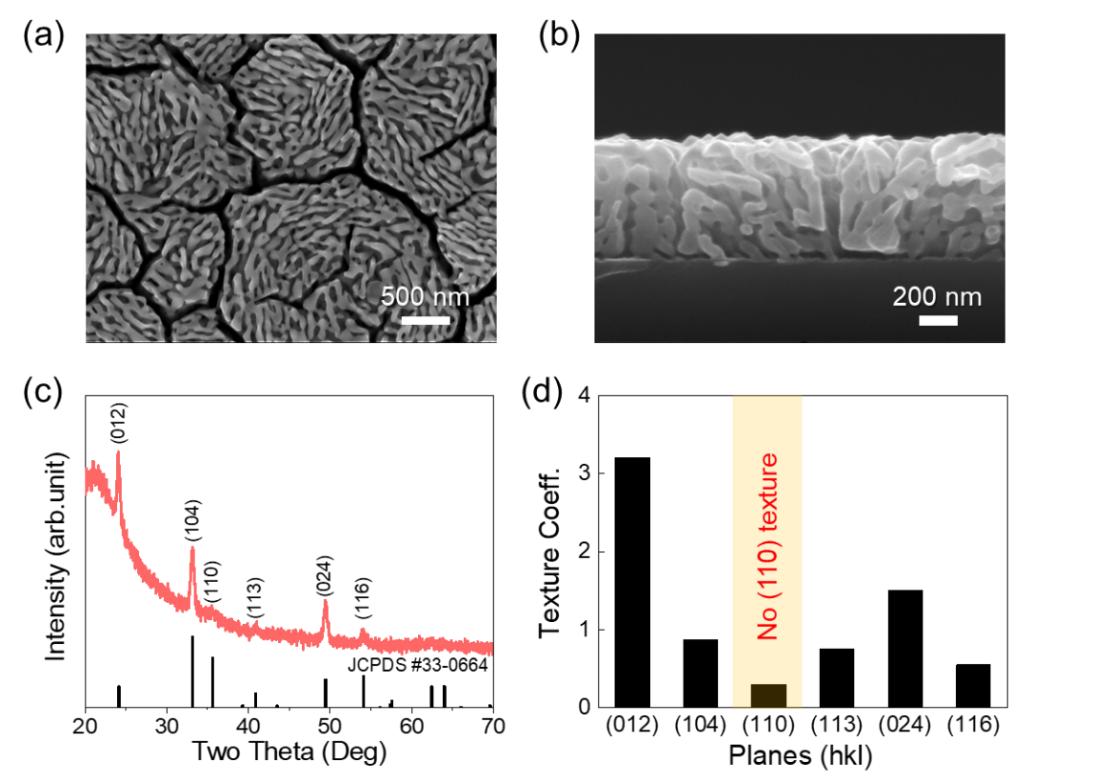


**Fig. S14** SEM and XRD analyses of tp-Fe_2_O_3_ grown on quartz substrate. **a** Top and **b** Cross-view SEM images. **c** XRD pattern. **d** TC analysis. For the quartz substrate, the highly porous morphology of Fe_2_O_3_ is maintained. However, no preferred texture (110) is observed


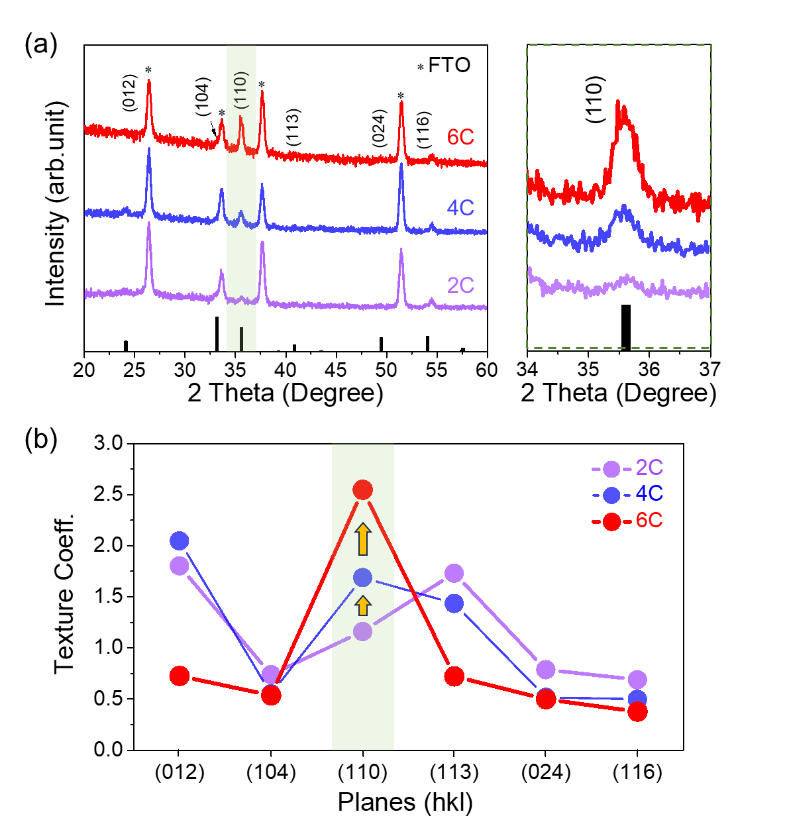


**Fig. S15** Texture evolution of tp-Fe_2_O_3_ grown on FTO substrate with varying growth cycles. **a** XRD patterns of tp-Fe_2_O_3_ synthesized with 2, 4, and 6 growth cycles. **b** TC analysis of different crystallographic planes, showing improved (110) orientation/texture with increasing growth cycles


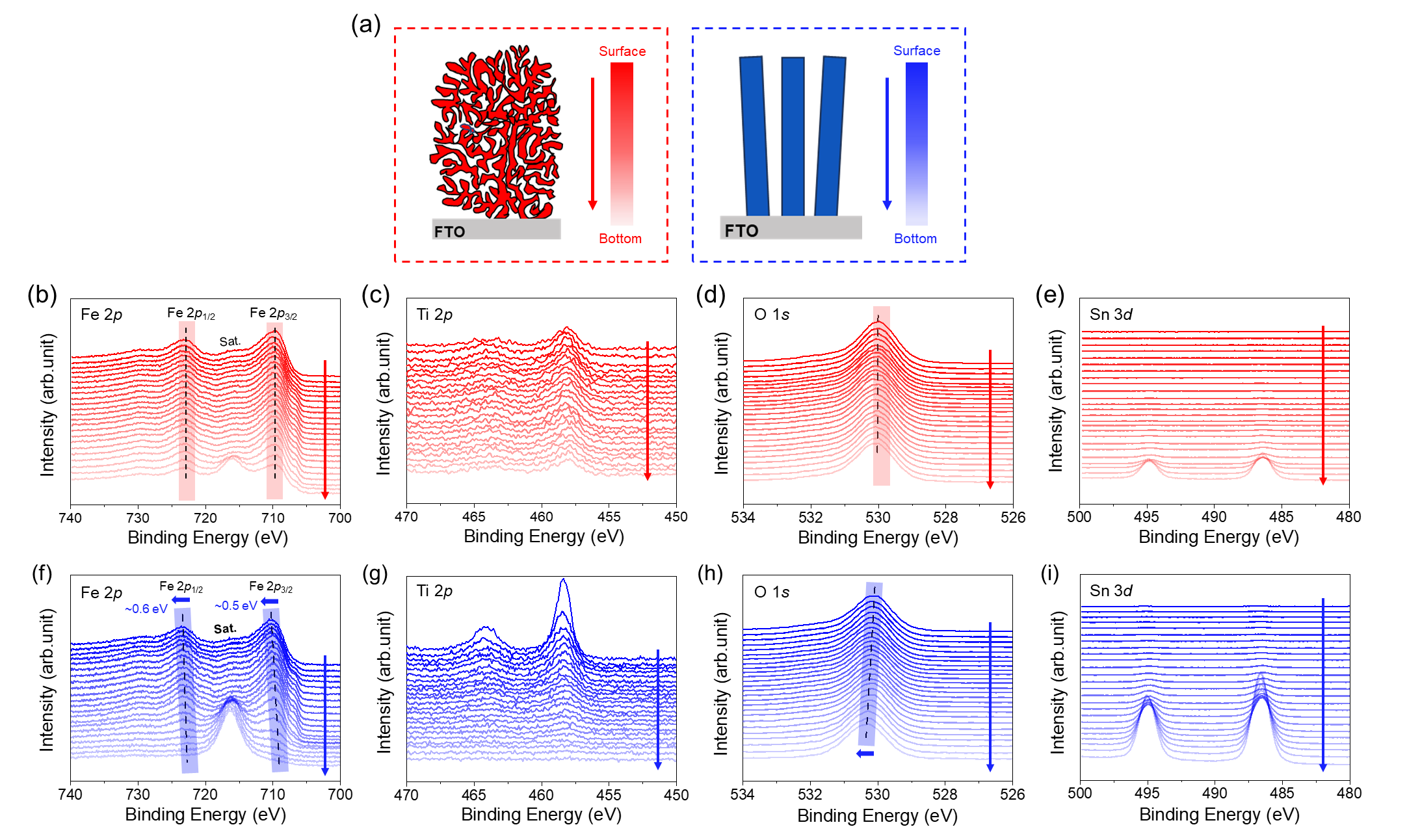


**Fig. S16** Depth-resolved XPS analysis with identical thickness: tp-Fe_2_O_3_ (red, top row) and nr-Fe_2_O_3_ (blue, bottom row). **a** Schematic illustrations showing the Ar⁺ ion etching direction for tp-Fe_2_O_3_ and nr-Fe_2_O_3_. **b**, **f** Fe 2p. **c**, **g** Ti 2p. **d**, **h** O 1s, and **e**, **i** Sn 3d spectra


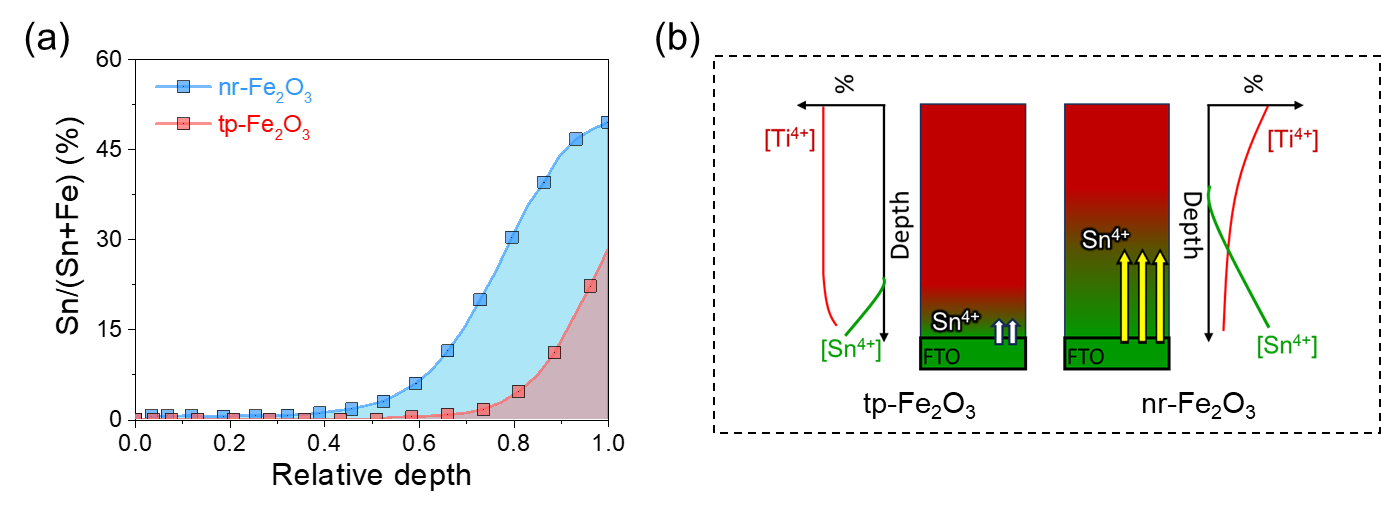


**Fig. S17** XPS depth profile analysis of Sn atomic concentration as a function of film depth. **a** Depth-dependent Sn/(Sn + Fe) atomic ratio profiles. **b** Schematic illustration of Ti and Sn dopant distributions. In tp-Fe_2_O_3_, Ti is homogeneously distributed, and Sn diffusion is minimized. In contrast, nr-Fe_2_O_3_ shows pronounced Sn diffusion and a less uniform Ti distribution. These results indicate that the in-situ Ti doping achieved via the MCFA method effectively limits Sn incorporation from the substrate, thereby reducing unintentional doping effects and preserving the conductivity of FTO.


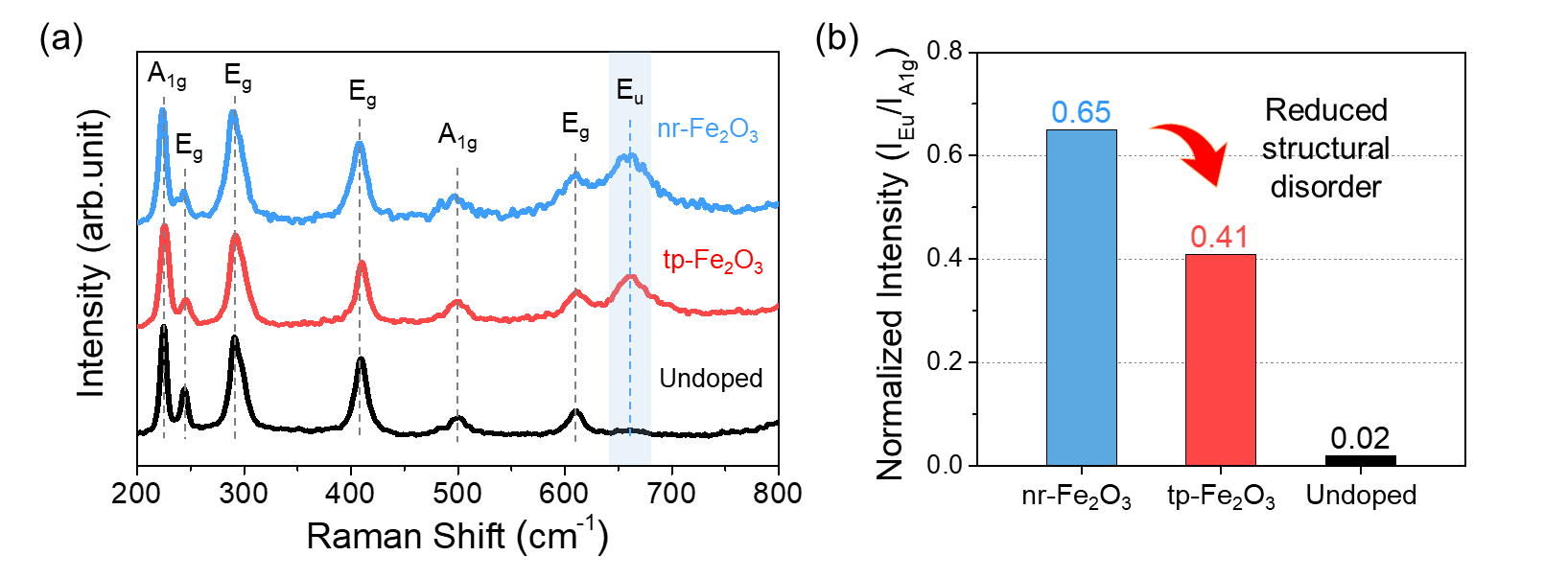


**Fig. S18** Raman analysis for tp-Fe_2_O_3_, nr-Fe_2_O_3,_ and control (undoped) Fe_2_O_3_. **a** Raman spectra. **b** Normalized Eu peak intensity (I_Eu_/I_A1g_)

**Discussion:** In nr-Fe_2_O_3_, the strong Eu Raman mode reveals symmetry breaking due to ex-situ doping, indicating abundant defects and oxygen vacancies that hinder charge transport. In contrast, tp- Fe_2_O_3_ shows a weaker Eu signal, consistent with uniform in-situ doping and preserved lattice symmetry.

A1g mode (~225 cm⁻¹) is a Raman-active symmetric stretching vibration of Fe–O bonds in α-Fe_2_O_3_. It is typically intense, consistent, and largely unaffected by minor structural distortions or doping, making it a reliable internal reference for comparing Raman spectra across different samples [S6]. In contrast, the Eu mode (~660–670 cm⁻¹) is infrared-active and Raman-inactive in ideal α-Fe_2_O_3_ with D3d symmetry. Its appearance in the Raman spectrum is attributed to symmetry breaking, oxygen vacancy formation, or dopant incorporation, all of which disrupt the ideal crystal lattice and activate this otherwise silent mode [S7].

In this study, the normalized Eu peak intensity (I_Eu_/I_A1g_) was used to quantify structural disorder. The tp-Fe_2_O_3_ sample (in-situ doped, hierarchical porous) shows lower disorder (0.41), whereas the nr-Fe_2_O_3_ sample (ex-situ doped, nanorod) exhibits higher disorder (0.65). This indicates that ex-situ doping in nr-Fe_2_O_3_ induces stronger symmetry breaking and a higher density of oxygen vacancies, consistent with its pronounced Eu Raman mode [S8].


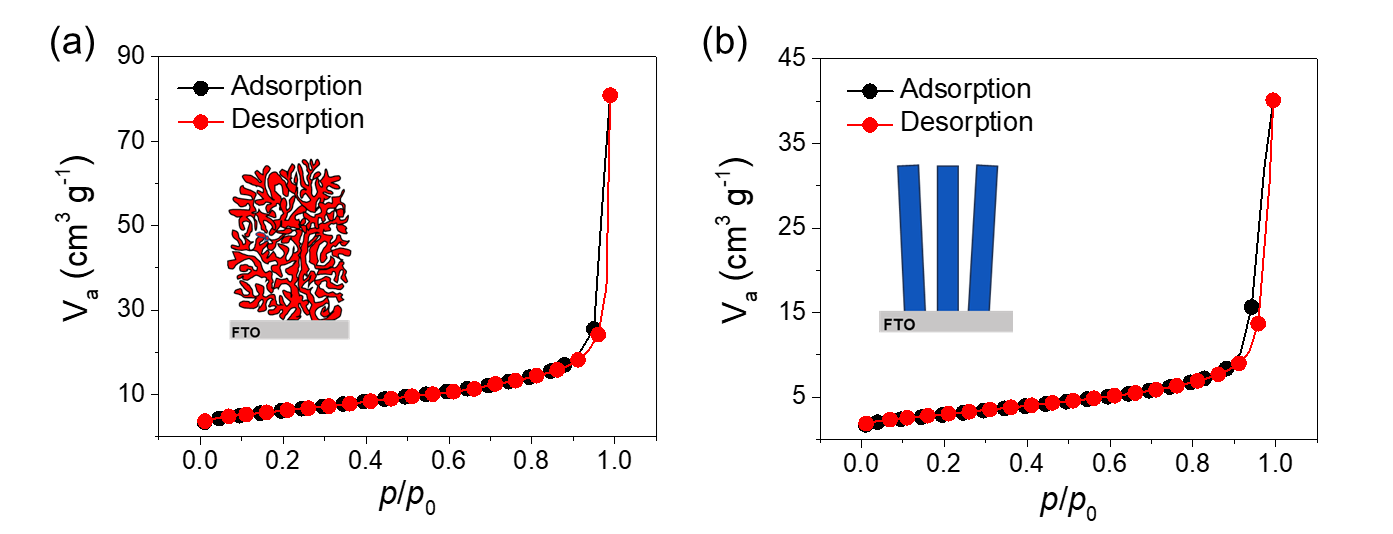


**Fig. S19** Brunauer-Emmett-Teller (BET) analysis. N_2_ adsorption–desorption isotherms curve for **a** tp-Fe_2_O_3_ and **b** nr-Fe_2_O_3_. Both film samples are scratched into the powder and collected for the BET analysis


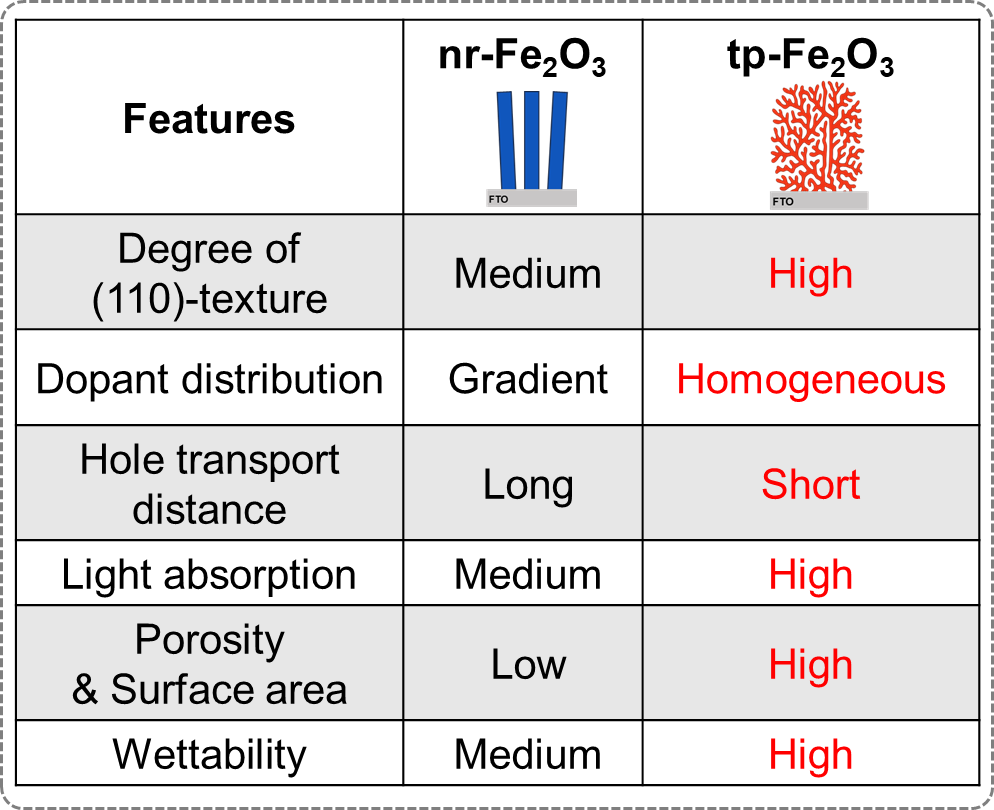


**Fig. S20** Summary of key distinguishing features of tp-Fe_2_O_3_ compared to nr-Fe_2_O_3_


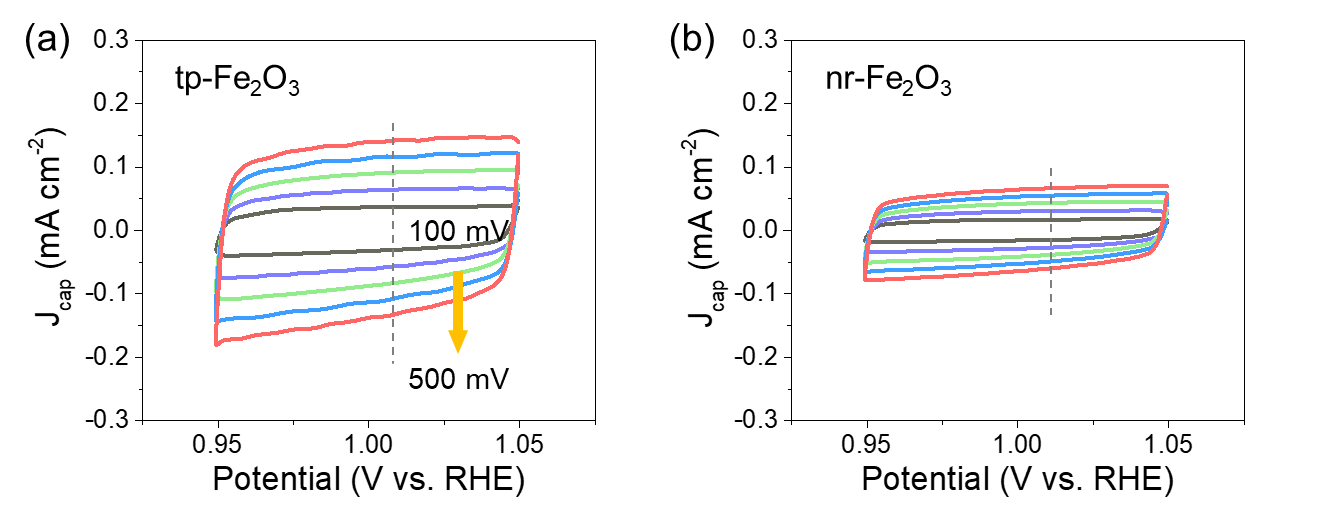


**Fig. S21** Capacitive current vs. potential curves measured in 1 M NaOH electrolyte. **a** tp-Fe_2_O_3_ and **b** nr-Fe_2_O_3_


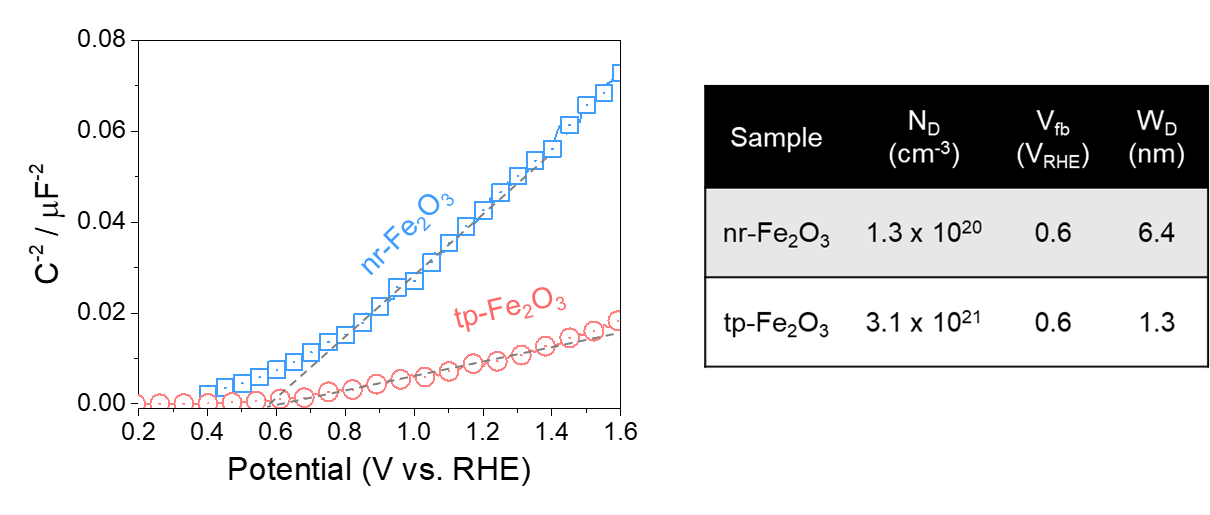


**Fig. S22** Mott-Schottky measurements of tp-Fe_2_O_3_ and nr-Fe_2_O_3_. The table displays the parameters for calculating the depletion region width (W_D_)


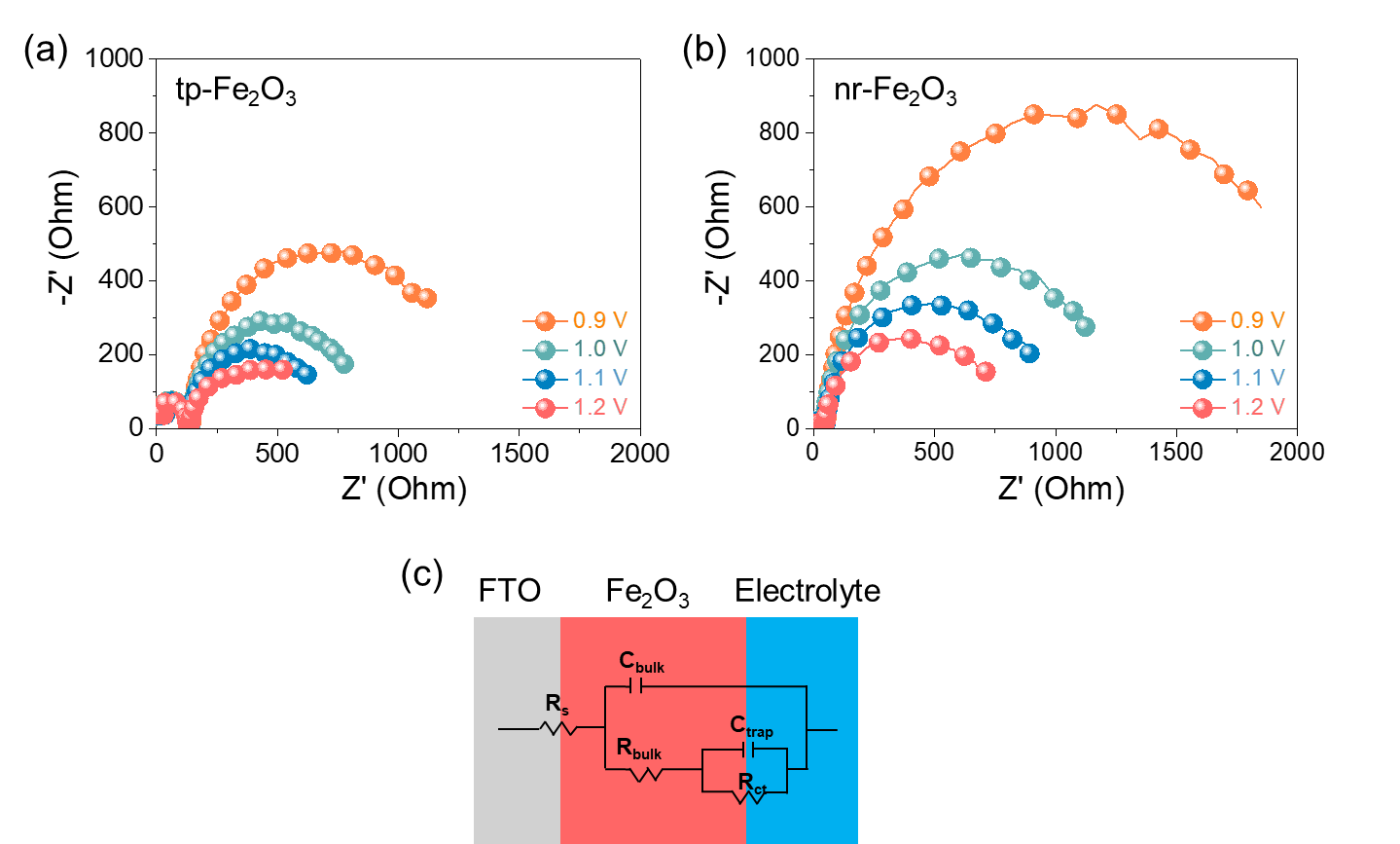


**Fig. S23** Potentiostatic electrochemical impedance spectroscopy (PEIS) analysis. **a** tp-Fe_2_O_3_, and **b** nr-Fe_2_O_3_ measured at different applied potentials (0.9-1.2 V). **c** The equivalent circuit model used to fit the EIS spectra


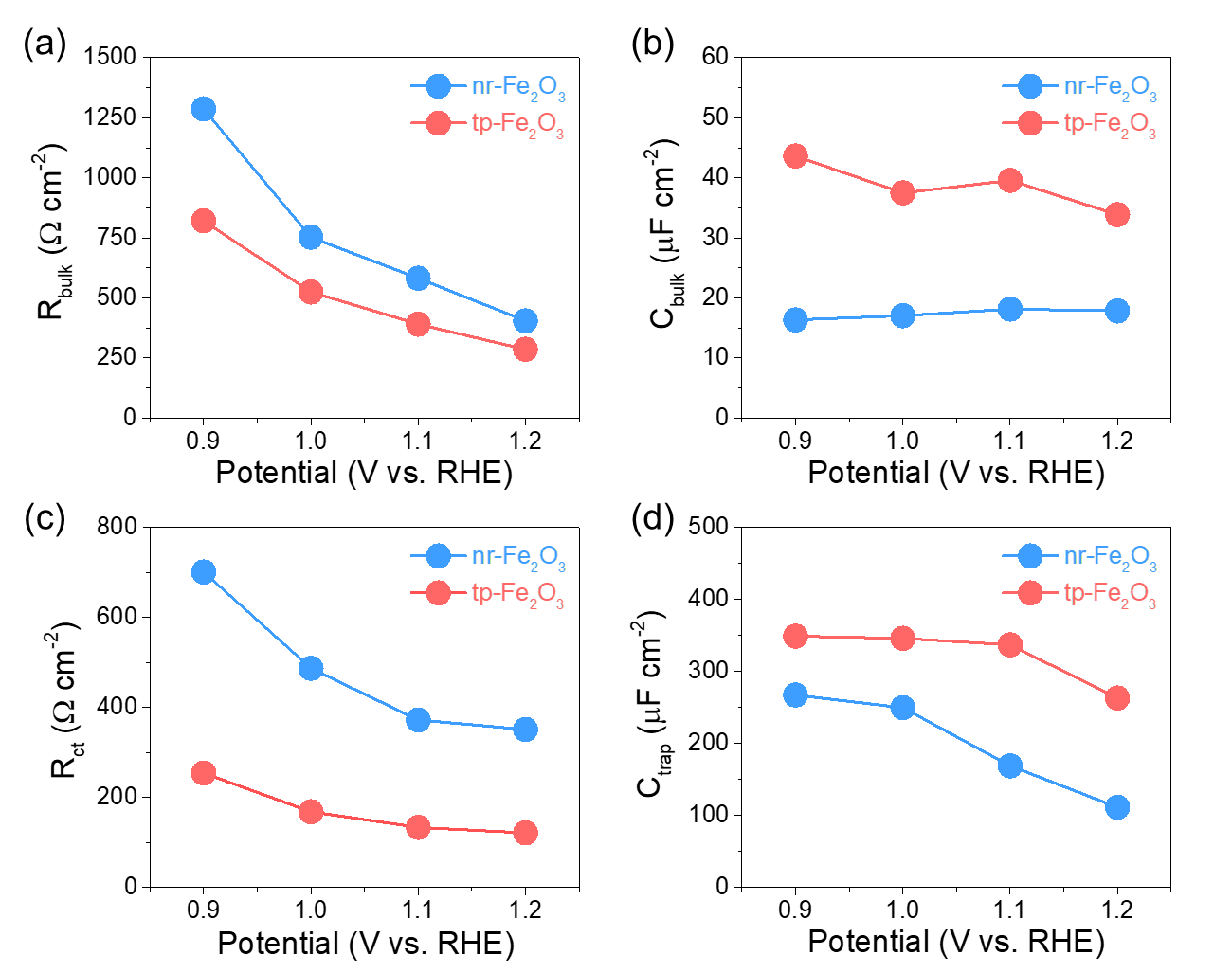


**Fig. S24** PEIS analysis. **a** R_bulk_ (resistance of hole trapping in the bulk), **b** C_bulk_ (capacitance of charge accumulation in the space charge layer), **c** R_ct_ (resistance between photoanode and electrolyte), and **d** C_trap_ (capacitance associated with charge accumulation on the surface states)


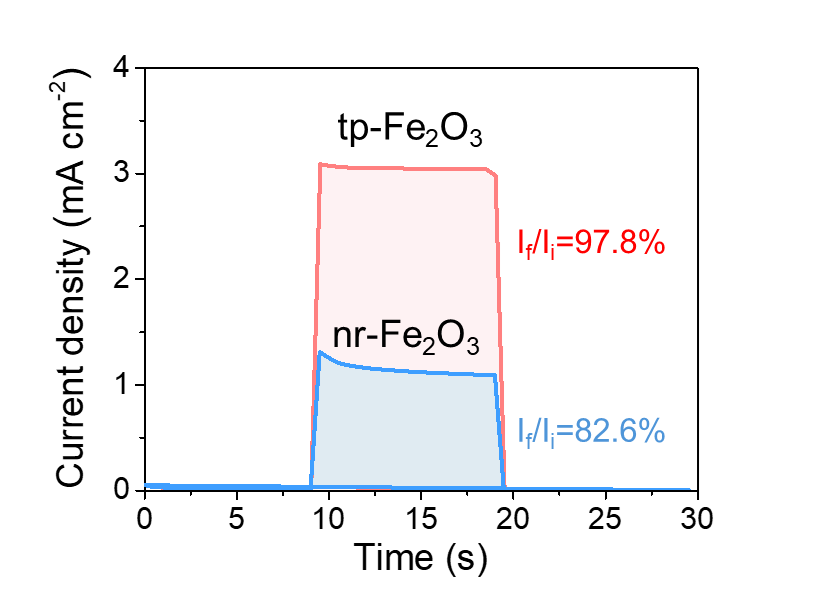


**Fig. S25** Transient current analysis of tp-Fe_2_O_3_ and nr-Fe_2_O_3_. I_i_ (initial) and I_f_ (final) represent the photocurrent densities immediately after light illumination and at steady state, respectively. The transient behavior reflects charge recombination characteristics, where a larger difference between I_i_ and I_f_ (i.e., lower I_f_/I_i_ ratio value) indicates higher charge recombination losses


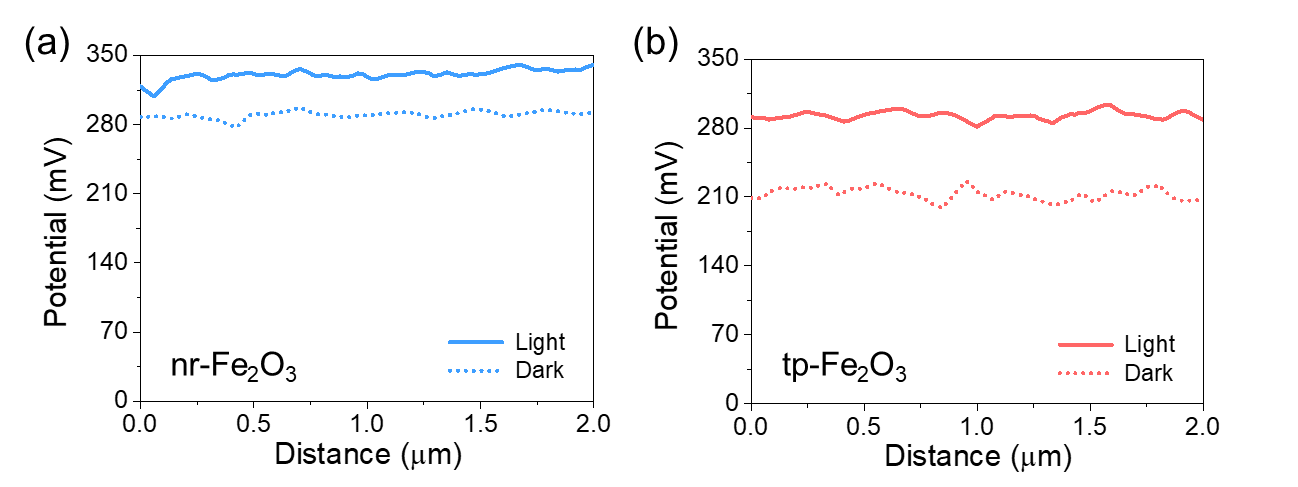


**Fig. S26** Kelvin probe force microscopy (KPFM) analysis of **a** nr-Fe_2_O_3_ and **b** tp-Fe_2_O_3_


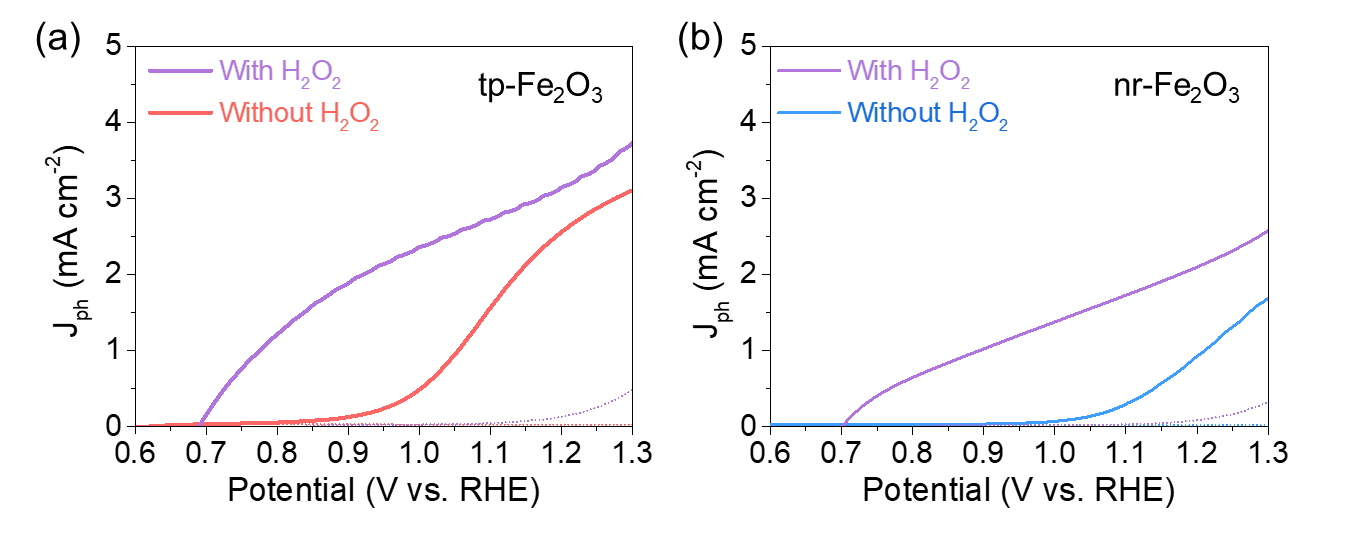


**Fig. S27** J-V curves measured with and without hole scavenger (H_2_O_2_). **a** tp-Fe_2_O_3_ and **b** nr-Fe_2_O_3_


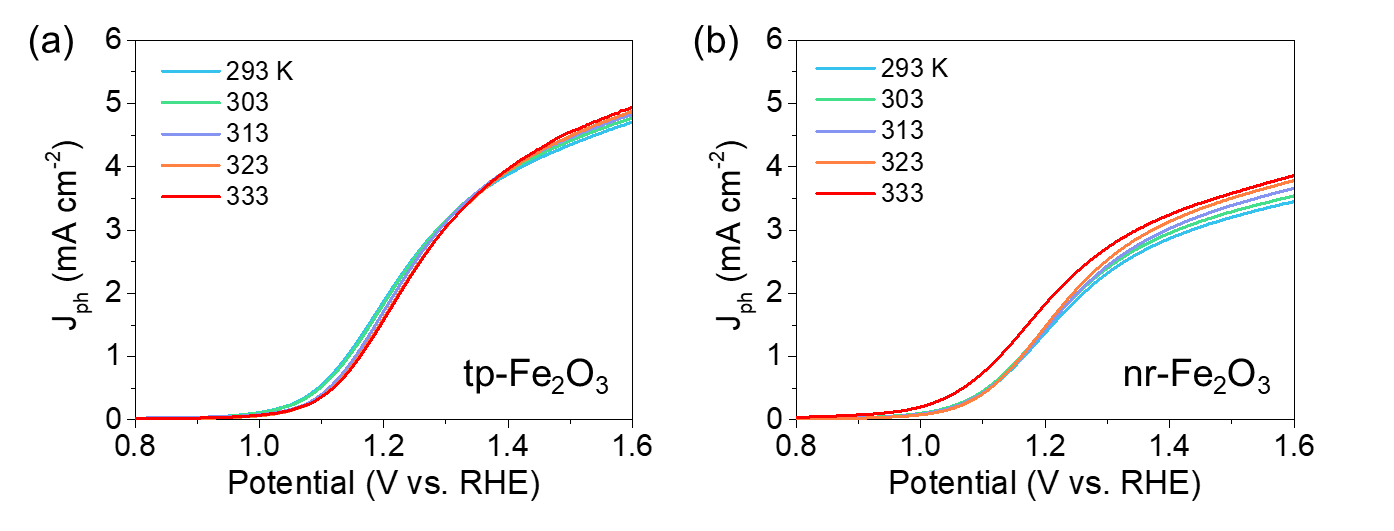


**Fig. S28** J-V curves measured under various temperatures for the calculation of the activation energy. **a** tp-Fe_2_O_3_ and **b** nr-Fe_2_O_3_

**
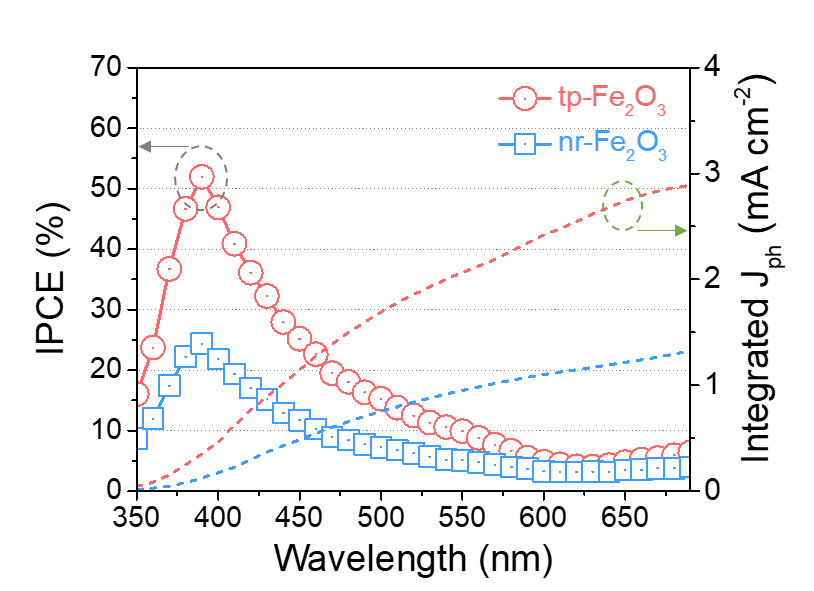
**

**Fig. S29** IPCE spectra of tp-Fe_2_O_3_ and nr-Fe_2_O_3_


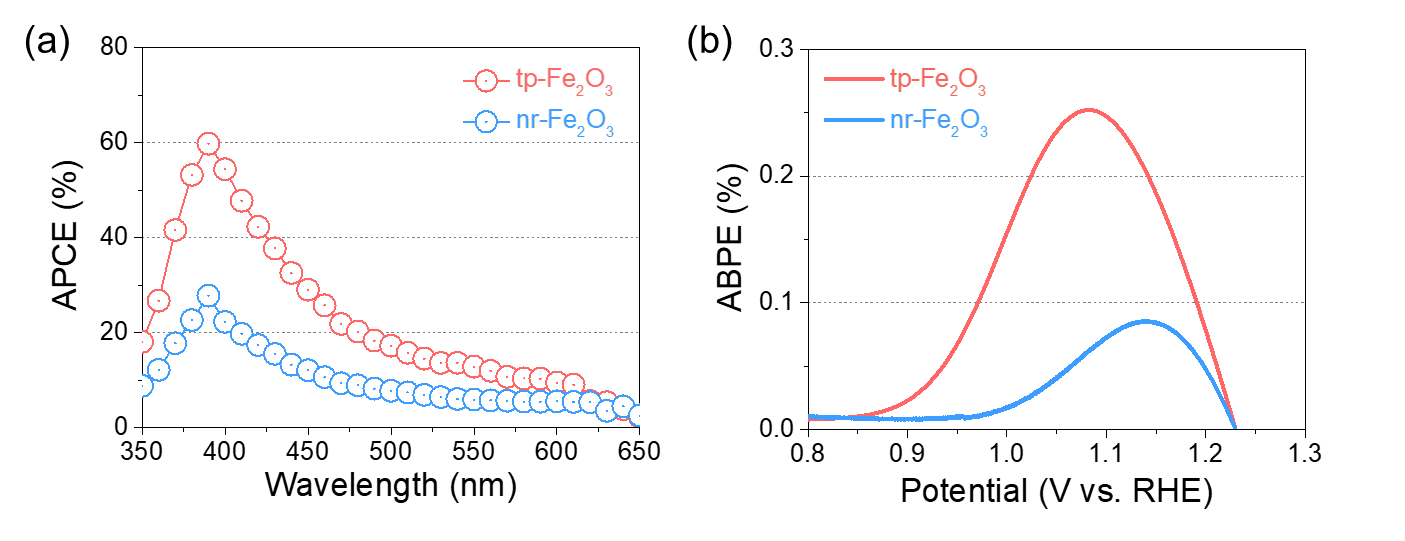


**Fig. S30 a** APCE spectra and **b** ABPE for tp-Fe_2_O_3_ and nr-Fe_2_O_3_


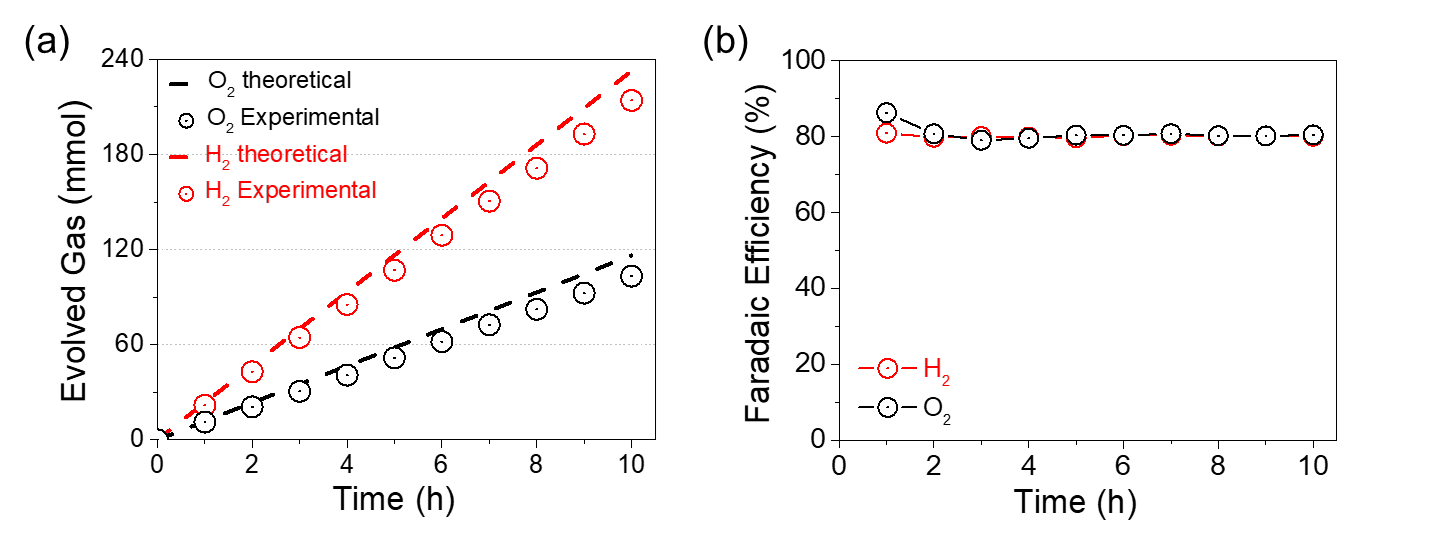


**Fig. S31** Gas evolution and Faradaic efficiency measurement of tp-Fe_2_O_3_ for OER. **a** H_2_/O_2_ production rate and **b** Faradaic efficiency


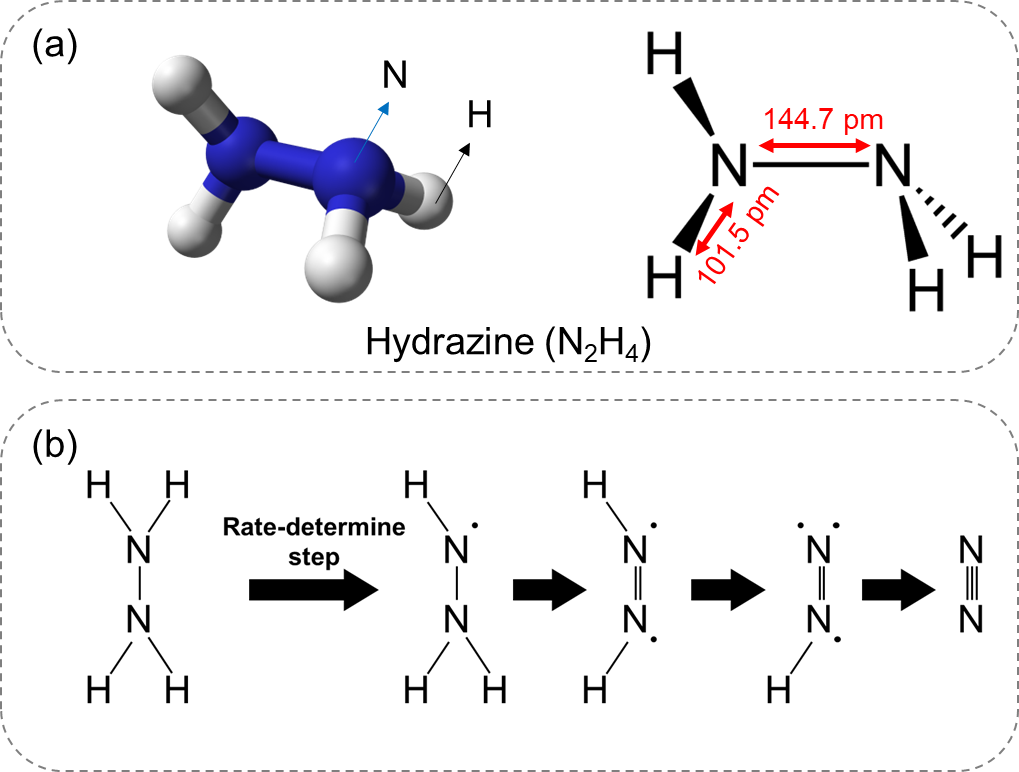


**Fig. S32** Molecular structure model and proposed reaction steps for PEC HzOR. **a** Molecular structure of N_2_H_4_. **b** The proposed reaction steps of PEC HzOR


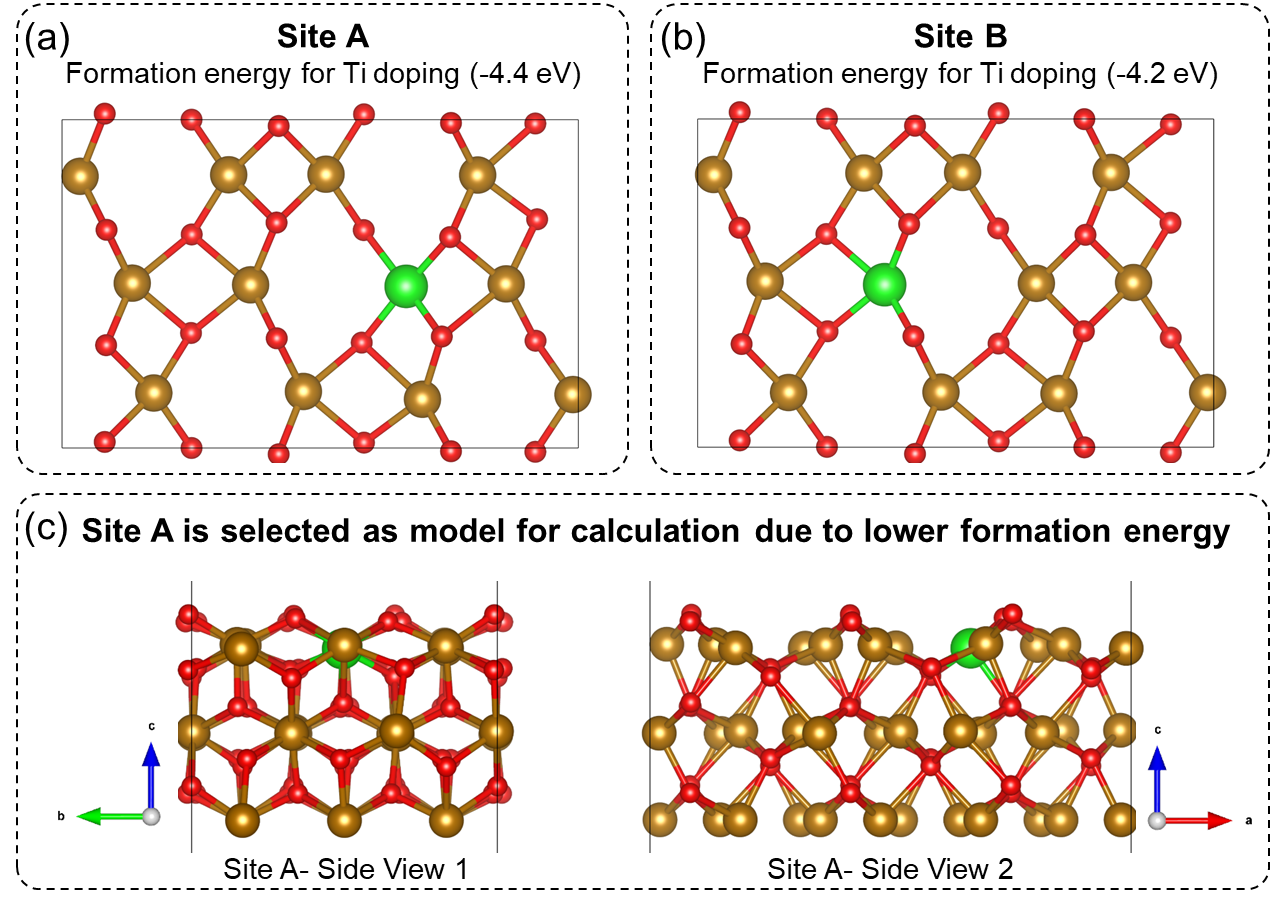


**Fig. S33** Ti substitution models on the Fe_2_O_3_ (110) surface: **a** site A and **b** site B (top views). Because site A has a lower formation energy, it is used for subsequent calculations. **c** The side view of the site A configuration. The Fe, O, and Ti atoms are denoted with brown, red, and green-colored spheres, respectively.

**Discussion:** DFT calculations were performed to elucidate the OER and HzOR mechanisms on the Ti‑doped Fe_2_O_3_ (110) surface. Among the two candidate substitutional sites examined, site A exhibited the lowest Ti formation energy, identifying it as the most favourable dopant position. Accordingly, the Fe_2_O_3_ (110) slab with Ti placed at site A was used for subsequent catalytic analysis.


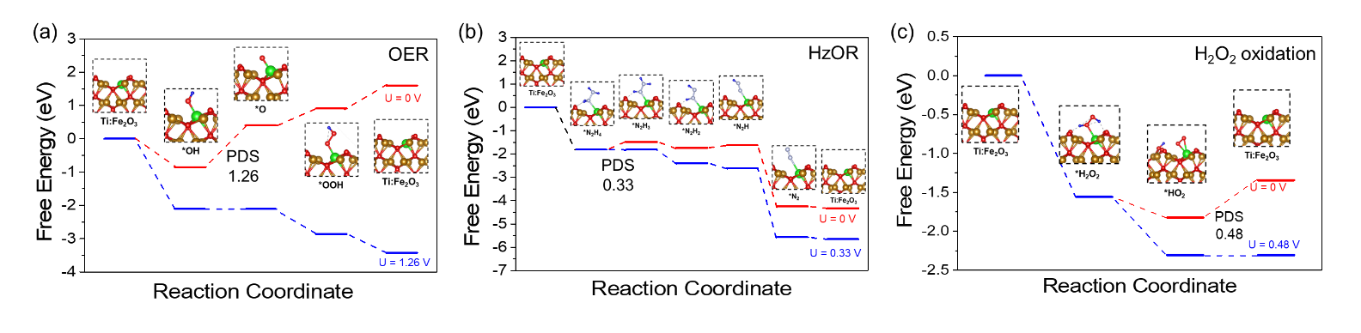


**Fig. S34** Comparison of free energy profiles for **a** OER and **b** HzOR. **c** H_2_O_2_ oxidation reaction.

**Discussion:** The adsorption energies of key intermediates involved in OER, HzOR, and H_2_O_2_ oxidation were calculated, and the corresponding free-energy diagrams in alkaline media were constructed. For the OER, the *OH → *O step was identified as the potential-determining step (PDS), with a thermodynamic energy barrier of 1.26 eV. In the case of H_2_O_2_ oxidation, the PDS requires an energy barrier of 0.48 eV. In contrast, the HzOR pathway is overall exothermic; its free-energy profile (evaluated at U = 0 V) exhibits a maximum uphill barrier of only 0.33 eV for the *N_2_H_4_ → *N_2_H_3_ step, significantly lower than the 1.26 eV required to drive all OER steps downhill, and even lower than that of H_2_O_2_ oxidation. These results indicate that HzOR is thermodynamically and kinetically favorable on Ti-doped Fe_2_O_3_ (110).


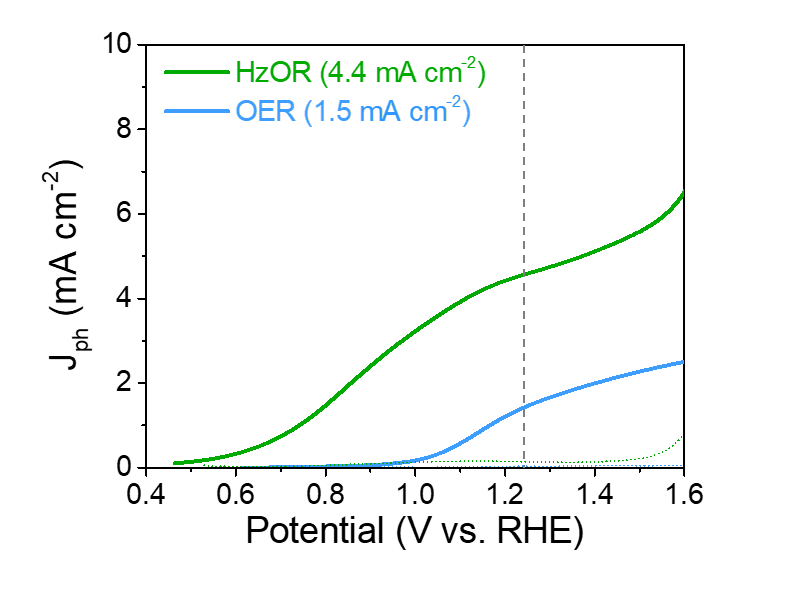


**Fig. S35** PEC OER and HzOR J-V curves of nr-Fe_2_O_3_


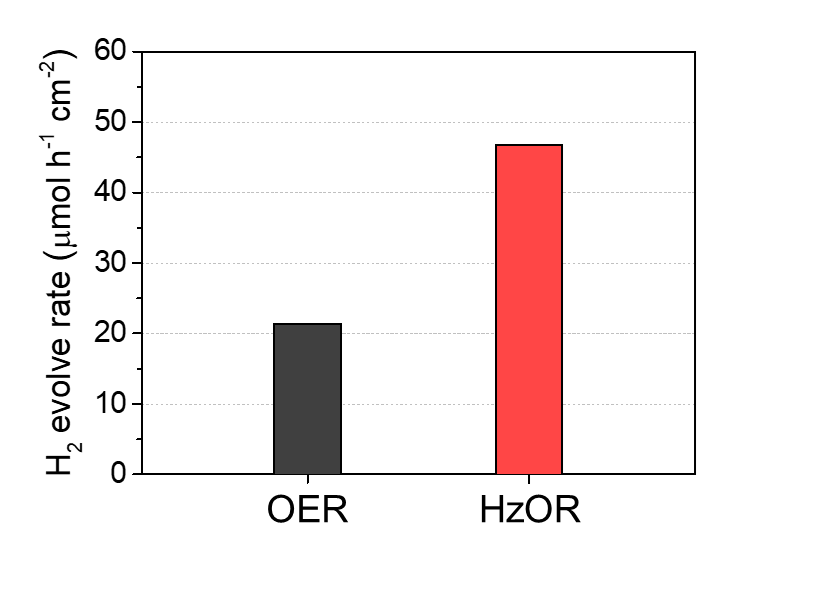


**Fig. S36** Comparison of H_2_ Production Rates via PEC HzOR and OER


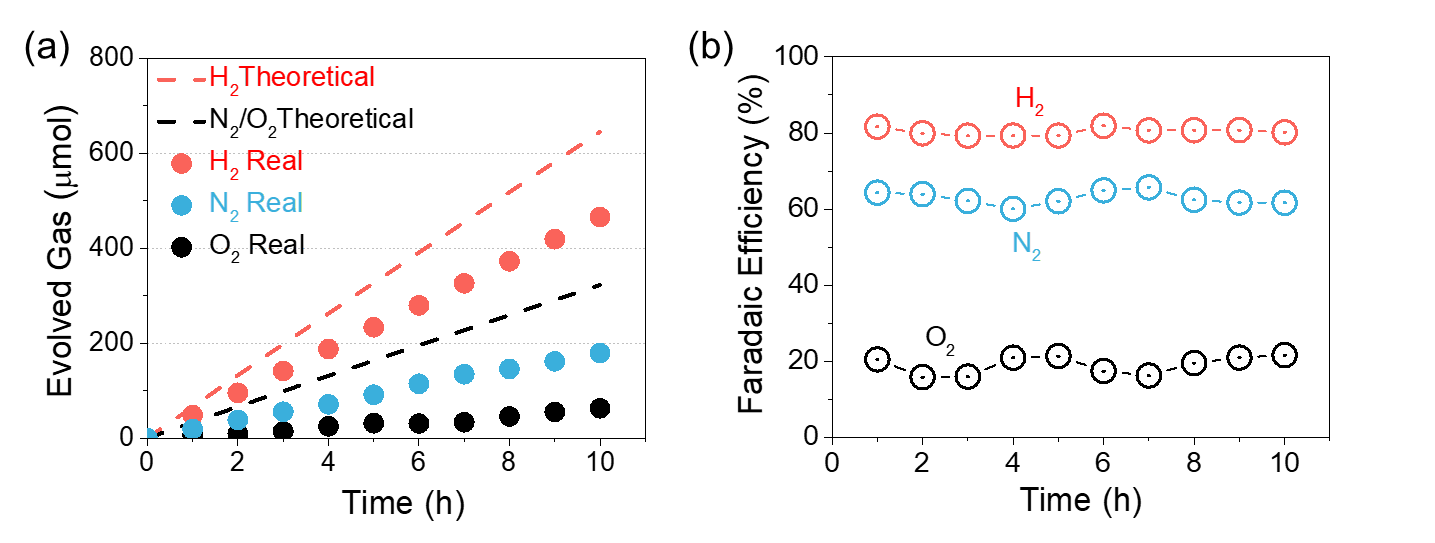


**Fig. S37 a** Gas evolution and **b** Faradaic efficiency of N_2_, O_2_, and H_2_ during PEC HzOR


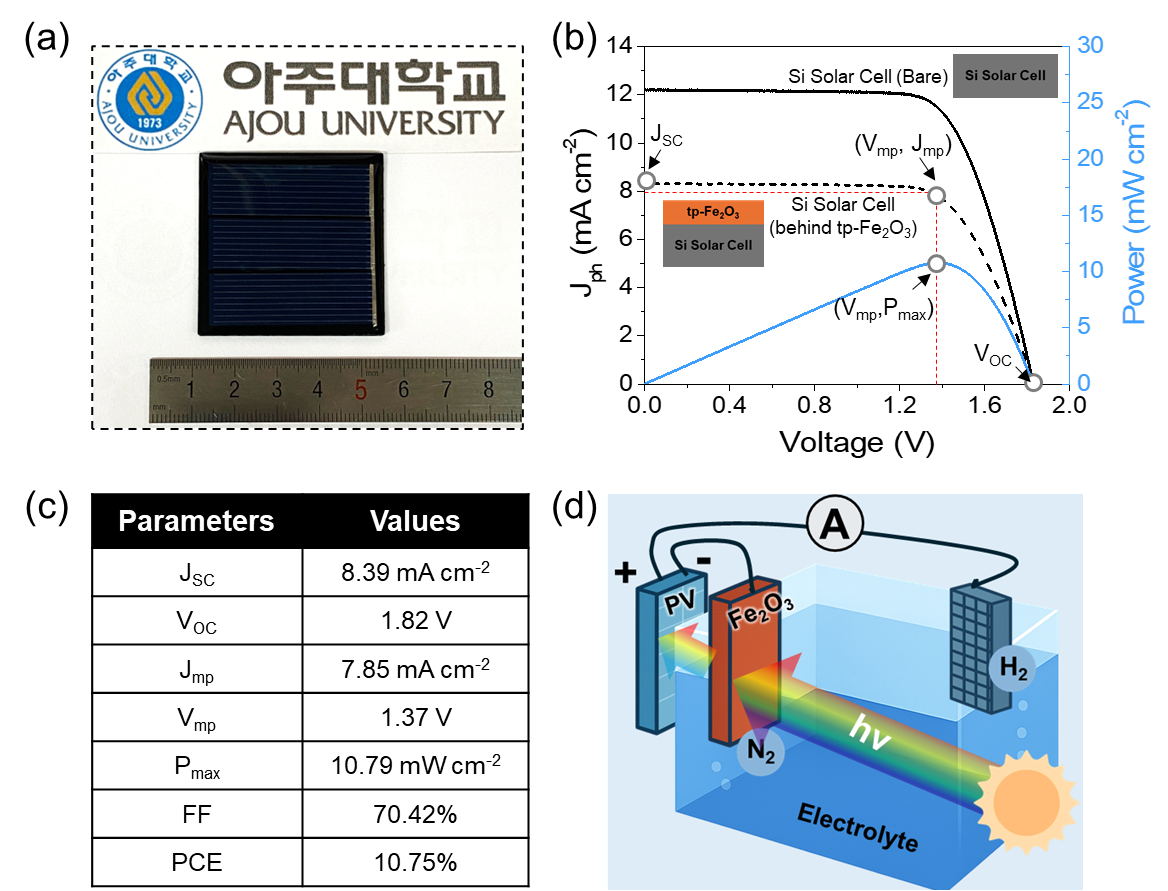


**Fig. S38** Bias-free H_2_ production demonstration via PV-PEC tandem system. **a** Photograph of the commercial c-Si solar cell module. **b** Current density–voltage (J–V) and power density–voltage (P–V) characteristics of a commercial crystalline silicon (c-Si) solar cell measured under 1-sun simulated illumination, either in the bare configuration or stacked behind the tp-Fe_2_O_3_ photoanode. The reduced photocurrent density observed in the stacked configuration is attributed to partial light absorption by the front tp-Fe_2_O_3_ layer. **c** Key performance parameters of the c-Si solar cell stacked behind the tp-Fe_2_O_3_ photoanode. **d** Schematic demonstration of PV-PEC device.

**Supplementary References**

1. G. Kresse, J. Furthmüller, Efficient iterative schemes for *ab initio* total-energy calculations using a plane-wave basis set. Phys. Rev. B **54**(16), 11169–11186 (1996). <https://doi.org/10.1103/physrevb.54.11169>
2. J.P. Perdew, K. Burke, M. Ernzerhof, Generalized gradient approximation made simple. Phys. Rev. Lett. **77**(18), 3865–3868 (1996). <https://doi.org/10.1103/physrevlett.77.3865>
3. P.E. Blöchl, Projector augmented-wave method. Phys. Rev. B **50**(24), 17953–17979 (1994). <https://doi.org/10.1103/physrevb.50.17953>
4. H.J. Monkhorst, J.D. Pack, Special points for Brillouin-zone integrations. Phys. Rev. B **13**(12), 5188–5192 (1976). <https://doi.org/10.1103/physrevb.13.5188>
5. J.K. Nørskov, J. Rossmeisl, A. Logadottir, L. Lindqvist, J.R. Kitchin et al., Origin of the overpotential for oxygen reduction at a fuel-cell cathode. J. Phys. Chem. B **108**(46), 17886–17892 (2004). <https://doi.org/10.1021/jp047349j>
6. D. Varshney, A. Yogi, Structural, vibrational and magnetic properties of Ti substituted bulk hematite: α-Fe_2–_*_x_*Ti x O3. J. Adv. Ceram. **3**(4), 269–277 (2014). <https://doi.org/10.1007/s40145-014-0117-x>
7. S.-H. Shim, T.S. Duffy, Raman spectroscopy of Fe_2_O_3_ to 62 GPa. Am. Mineral. **87**(2–3), 318–326 (2002). <https://doi.org/10.2138/am-2002-2-314>
8. K.-Y. Yoon, J. Park, M. Jung, S.-G. Ji, H. Lee et al., NiFeOx decorated Ge-hematite/perovskite for an efficient water splitting system. Nat. Commun. **12**, 4309 (2021). <https://doi.org/10.1038/s41467-021-24428-7>
